# Fungal Signature of Moisture Damage in Buildings: Identification by Targeted and Untargeted Approaches with Mycobiome Data

**DOI:** 10.1128/AEM.01047-20

**Published:** 2020-08-18

**Authors:** Rachel I. Adams, Iman Sylvain, Michal P. Spilak, John W. Taylor, Michael S. Waring, Mark J. Mendell

**Affiliations:** a Plant and Microbial Biology, University of California, Berkeley, California, USA; b California Department of Public Health, Richmond, California, USA; c Civil, Architectural and Environmental Engineering, Drexel University, Philadelphia, Pennsylvania, USA; University of Manchester

**Keywords:** dampness, dust, fungi, growth requirements, indoor, mold, water damage

## Abstract

Identifying microbial indicators of damp and moldy buildings remains a challenge at the intersection of microbiology, building science, and public health. Sixty homes in New York City were assessed for moisture-related damage, and three types of dust samples were collected for microbiological analysis. We applied four approaches for detecting fungal signatures of moisture damage in these buildings. Two novel targeted approaches selected specific taxa, identified by *a priori* hypotheses, from the broad mycobiome as detected with amplicon sequencing. We investigated whether (i) hydrophilic fungi (i.e., requiring high moisture) or (ii) fungi previously reported as indicating damp homes would be more abundant in water-damaged rooms/homes than in nondamaged rooms/homes. Two untargeted approaches compared water-damaged to non-water-damaged homes for (i) differences between indoor and outdoor fungal populations or (ii) differences in the presence or relative abundance of particular fungal taxa. Strong relationships with damage indicators were found for some targeted fungal groups in some sampling types, although not always in the hypothesized direction. For example, for vacuum samples, hydrophilic fungi had significantly higher relative abundance in water-damaged homes, but mesophilic fungi, unexpectedly, had significantly lower relative abundance in homes with visible mold. Untargeted approaches identified no microbial community metrics correlated with water damage variables but did identify specific taxa with at least weak positive links to water-damaged homes. These results, although showing a complex relationship between moisture damage and microbial communities, suggest that targeting particular fungi offers a potential route toward identifying a fungal signature of moisture damage in buildings.

**IMPORTANCE** Living or working in damp or moldy buildings increases the risk of many adverse health effects, including asthma and other respiratory diseases. To date, however, the particular environmental exposure(s) from water-damaged buildings that causes the health effects have not been identified. Likewise, a consistent quantitative measurement that would indicate whether a building is water damaged or poses a health risk to occupants has not been found. In this work, we tried to develop analytical tools that would find a microbial signal of moisture damage amid the noisy background of microorganisms in buildings. The most successful approach taken here focused on particular groups of fungi—those considered likely to grow in damp indoor environments—and their associations with observed moisture damage. With further replication and refinement, this hypothesis-based strategy may be effective in finding still-elusive relationships between building damage and microbiomes.

## Abstract (precis)

Living or working in damp or moldy buildings increases the risk of many adverse health effects, including asthma and other respiratory diseases. To date, however, the particular environmental exposure(s) from water-damaged buildings that causes the health effects have not been identified. Likewise, a consistent quantitative measurement that would indicate whether a building is water damaged or poses a health risk to occupants has not been found. In this work, we tried to develop analytical tools that would find a microbial signal of moisture damage amid the noisy background of microorganisms in buildings. The most successful approach taken here focused on particular groups of fungi—those considered likely to grow in damp indoor environments—and their associations with observed moisture damage. With further replication and refinement, this hypothesis-based strategy may be effective in finding still-elusive relationships between building damage and microbiomes.

## INTRODUCTION

Although there are well-established links between spending time in a damp and moldy building and adverse health effects (1, 2), we still do not know how to make microbial measurements that can reliably identify damp and moldy buildings. Studies using a variety of techniques have reported that damaged homes are linked, variously, to increases in the fungal genera *Penicillium* and *Aspergillus* (3–5), to increases in other taxonomic groups (6, 7), to changes in indoor-outdoor microbial differences (8–11), or not consistently to any microbiological measurements (12–14).

Amid these and other efforts, there remains a strong need to identify microbiological measurements in buildings that are reliably correlated with the presence of dampness and mold. Currently, qualitative or semiquantitative observational indicators (e.g., visible mold, mold odor, moisture, and moisture damage) have shown the strongest associations with health outcomes (15). However, identifying quantitative microbiological measurements that correlate even more strongly with building moisture or the resulting health effects would offer several advantages. Quantitative microbial metrics would support the development of standardized techniques to identify moisture problems in buildings, even if hidden. Also, identifying particular microbial agents associated with dampness and mold would allow exploration of the biological mechanisms, still not entirely understood, involved in dampness-related health effects in building occupants.

The difficulties to date in detecting a consistent microbial signal of moisture damage could be largely due to limitations in sampling and identification of microbes. For sampling, researchers now may collect particles previously airborne, where the particles settled on passive deposition samplers set out for a known time period or were captured on a filter by active sampling over a known time interval. They may also sample particles that have settled during an indefinite period by vacuuming floors or mattresses (16). However, it is not clear how well these different sampling strategies capture true airborne concentrations resulting from damp and moldy building materials. Regarding limitations in identification, until relatively recently, researchers characterized microorganisms through culture, spore counts, or chemical constituents (endotoxin, ergosterol, etc.). This limited picture of the overall taxonomic composition was broadened with the advent of DNA sequence-based tools to both identify specific microbes and characterize entire microbiomes. However, while current methods may better capture the broad microbial community, the resolution of the taxonomic identifications can vary. These limitations may explain why signals of water damage detected by earlier microbiome analyses have been subtle (7, 11), especially considering the variation in the microbiomes of buildings across time, space, building materials, and construction styles (17).

Here, we compare different methods of both sampling and analyzing the microbiomes of buildings, seeking approaches that best identify a fungal signature of moisture. In a study of homes in New York City, we applied two general types of approaches: targeted approaches, focusing on specific taxa with previously determined characteristics that, *a priori*, make them promising indicators of damp building conditions, and untargeted approaches, using statistical analyses to identify associations with either community characteristics or specific taxa, without *a priori* targets. The aim of our research is to identify promising approaches that could then be applied to other data sets, with the goal of developing broadly applicable, quantitative microbiological assessments that reliably characterize dampness and associated mold growth in buildings.

## RESULTS

### Overview of study homes.

Data collection for the first sampling period, in January (dust sampling, measurements, researcher inspection, and occupant surveys), was completed in 59 homes. For the second sampling period, in August (dust sampling and occupant surveys only), 45 of these 59 homes participated. Although 10 new homes were added in August, these were not included in the current analyses because they lacked researcher inspection for moisture or mold damage. Thus, contemporaneous researcher inspection and environmental sampling occurred in 59 homes, and for 45 of those homes, microbial profiles were also available 6 months after the initial researcher inspection.

**FIG 1:**
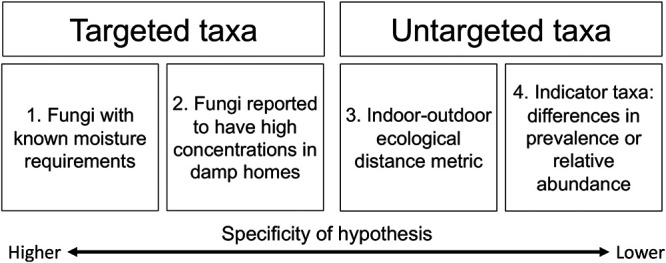
The four different approaches used to explore a fungal signature of moisture damage. Approaches 1 and 2 target particular taxa in the fungal community, while 3 and 4 consider the fungal community more broadly in an untargeted fashion. The specificity of the hypothesis decreases from approach 1 through approach 4.

We report our results for each of four analytical approaches, using two methods that targeted specific taxa within the mycobiome data and two that considered the entire mycobiome (Fig. 1).

### Approach 1: fungi with known moisture requirements.

Normal building environments are too dry to support growth, and fungi need elevated moisture to grow indoors. Moisture available to support microbial growth is measured as the water activity (a_w_, unitless, range 0 to 1) of the substrate, defined as the vapor pressure of the water in the material as a fraction of the saturation vapor pressure (18). Those fungi that need the least elevation in moisture, the xerophiles, can grow even on substrates with only slightly elevated water activity (generally, 0.65 < a_w_ < 0.80). Hydrophiles are fungi that grow only with high water availability (a_w_ ≥ 0.90), while mesophiles require intermediate levels. In approach 1 analyses, we compared, across levels of observed dampness, the total counts found of each of the three sets of fungi categorized by moisture requirements: hydrophilic, mesophilic, and xerophilic. We hypothesized that hydrophilic fungi may be more abundant in water-damaged rooms/homes than in nondamaged rooms/homes. However, we note that the type of fungi predominating in damp buildings would likely depend on the extent and time course of moisture failures in those buildings. For instance, moisture could be elevated slightly, to levels at which only xerophiles could proliferate, but not high enough for mesophiles and hydrophiles to grow. Likewise, the elevated moisture with increasing proximity to a plumbing leak might support xerophiles, then mesophiles, then hydrophiles, sequentially, or leaks, after their initiation, might over time support these fungal groups in the same sequence as moisture accumulates. Although lacking specific information on these aspects of the moisture failures in the buildings studied, we assessed the three fungal groups separately in the analyses to look for any consistent patterns of proliferation with moisture failures generally. The specific fungal species targeted and their moisture requirements for growth are provided as Data Set S1 in the supplemental material.

Given that assignment of taxonomy is critical in this approach that relies on species-level classification, we compared taxonomic identifications of the fungal amplicon sequence variants (ASVs) between two UNITE fungal reference databases that handle taxonomic singletons differently, one including all global taxonomic singletons and another including only singletons manually curated by taxonomic experts, known as reference singletons (19). We refer to these two distributions of the reference databases as UNITE with global singletons and UNITE with reference singletons. Of the 108 species for which moisture requirements for growth were compiled from the literature, 91 were present in one or both of the UNITE databases. Thus, 91 is the maximum number of species that could be present in a reduced data set of observed fungi with known moisture requirements, based on our compiled list and the version of the UNITE database used. We observed 60 species when taxonomy was assigned with the UNITE global singletons fungal database (Table 1). This reduced data set represented a small portion of the entire mycobiome data. For example, the hydrophilic fungi included made up approximately 3% of the counts in the full assessed community. An individual species may have been represented by many different fungal ASVs, as the 60 identified species with known moisture requirements were represented by a total of 627 ASVs. The results when taxonomy was assigned with the UNITE reference singletons were, overall, similar to the taxonomic assignments made with the UNITE global singletons, and a comparison between the two taxonomic assignments is included in File S1 in the supplemental material.

**TABLE 1:** Representation in our data set of fungi either with known moisture requirements for growth or included in group 1 ERMI fungi using the UNITE fungal database with global singletons

Fungal group	Moisture requirements for growth	No. of taxa identified in the literature	No. of those taxa identified in this dataset	% of total sequences in this dataset
Hydrophilic	≥0.90 a_w_	18	7	3.2
Mesophilic	0.80 ≤ a_w_ < 0.90	61	35	19.6
Xerophilic	<0.80 a_w_	29	18	5.6
Group 1		43	25	8.5

Reporting of results for approach 1 will focus on analyses using the relative abundances of taxa, taxonomic assignment based on the fungal reference database with global singletons, and contemporaneous building damage assessments and microbiological measurements, with brief mention of differences in the other analyses. Full results of all analyses are provided in the supplemental material.

**FIG 2:**
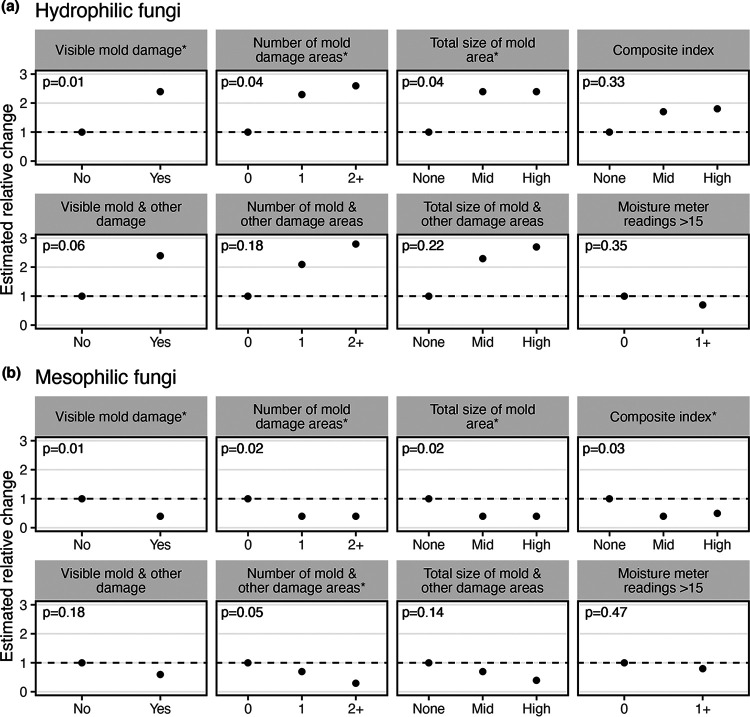
Hydrophilic and mesophilic fungi in vacuumed floor dust. Estimated relative changes in the relative abundances of hydrophilic (a) and mesophilic (b) fungi in vacuumed floor dust of homes across various indicators of moisture damage. The estimated relative change was determined using the negative binomial model estimate relative to the “no damage” category, indicated here as a dashed line at a value of 1.

For dust in vacuum samples, the relative abundances of hydrophilic fungi (growth requirements of ≥0.90 a_w_) were consistently 2 to 3 times greater in homes with observed mold or other damage than in homes without, with negative binomial-model *P* values of <0.05 for three of the eight damage metrics analyzed (Fig. 2a and Table S1). Mesophilic fungi (0.80 ≤ a_w_ < 0.90) in vacuum samples unexpectedly showed strong opposite trends: the relative abundances in homes with visible mold were lower, by approximately half, than those in homes without visible mold (*P* values of <0.05 for five of the eight metrics analyzed) (Fig. 2b and Table S1). Xerophilic fungi (<0.80 a_w_) in vacuum samples showed only weak, nonsignificant associations in both directions (Table S1). Homes with moisture meter readings of >15 showed no clear fungal associations in these or most other analyses performed.

For dust fall collector samples (Table S1), hydrophilic fungi showed trends similar to those in vacuum samples, some with even stronger associations, with *P* values of <0.05 for two of eight damage metrics. Mesophilic and xerophilic fungi showed no clear associations in dust fall collector samples.

For door trim swab samples, we compared fungal groups of known moisture requirements across levels of damage separately for specific room types and for entire homes (Table S2). Rooms with both the door trim swab samples and damage assessments needed for these comparisons were mostly bedrooms (59%) and bathrooms (39%). In door trim swab samples, unlike in vacuum and dust fall collector samples, xerophilic fungi had statistically significant positive associations with all damage metrics, a pattern seen strongly in room-specific analyses but more weakly with home-level analyses. In contrast, in door trim swab samples taken 6 months after a building assessment, xerophilic fungi had much lower relative abundances in these rooms. For hydrophilic and mesophilic fungi, few associations were apparent other than a slight tendency for increased mesophiles in damaged rooms.

The fungal database chosen to assign taxonomy to sequences, whether with global singletons or reference singletons, influenced the magnitudes of the estimated relative changes and the *P* values of the statistical models, but not the trends observed (Table S3). Absolute abundances of fungi (i.e., relative abundance adjusted for total fungal biomass as assessed with quantitative PCR [qPCR]) compared with relative abundances showed slightly different patterns (Table S4). For absolute abundance, hydrophilic fungi in vacuum samples tended to be substantially less abundant, in contrast to the results for relative abundance. Also, based on absolute abundance, mesophilic fungi in both vacuum samples and dust fall collector samples showed decreased abundance in homes with visible mold damage, while based on relative abundance, mesophilic fungi showed decreased abundance only in vacuum dust samples of these homes. Although with absolute abundance, hydrophilic fungi in dust fall collector samples were much more abundant in homes with visible mold and other types of damage, these relationships were driven entirely by a single home. Finally, for fungal assessment conducted 6 months after the building damage assessment, xerophilic fungi in dust fall collector samples showed a tendency to have greater relative abundance in homes previously reported to have visible mold and damage than in homes without damage (data not shown).

### Approach 2: group 1 ERMI fungi.

Vesper et al. (20) reported that a set of fungal taxa, as measured by qPCR assays, were found at higher levels in water-damaged than in non-water-damaged homes. The combined concentrations of these taxa (group 1), adjusted for the combined concentrations of other fungal taxa reported to be unrelated to home dampness (group 2), constitute the environmental relative moldiness index (ERMI). This metric has been recommended for indicating water damage, even if hidden, in homes (21). The full list of ERMI species has been published (see Table 1 and footnotes in reference 22) and is presented here, with current taxonomy, in Data Set S2. The current analyses focused on the ERMI group 1 taxa (20 assays, 43 currently identified taxa) as taxa reported to be associated with water damage in buildings. We hypothesized that this group would have increased abundance in damp homes.

Of the 43 group 1 fungal taxa, 25 were identified in the mycobiome data where taxonomy was assigned with global singletons, representing over 8% of the total sequences (Table 1). Of the 43 group 1 fungi, 26 overlapped with those fungi targeted in approach 1 for their specific moisture requirements. Of these, only four taxa were classified as hydrophilic, while eight were mesophilic and over half (*n* = 14) were xerophilic.

**FIG 3:**
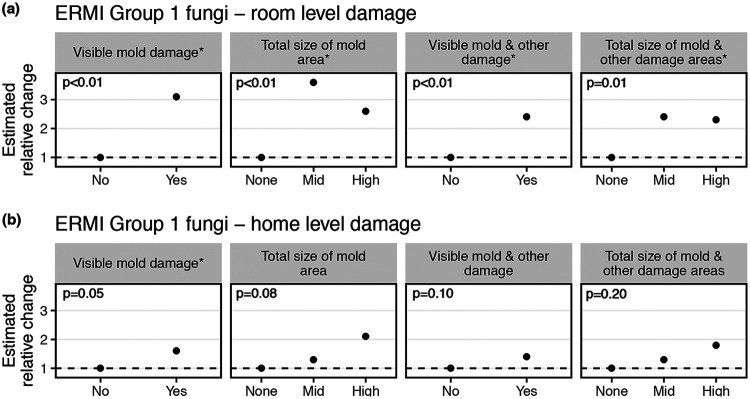
ERMI group 1 fungi in the door trim swab samples. Estimated relative changes in the relative abundances of ERMI group 1 fungi in the door trim swab samples of rooms (a) and houses (b) across various indicators of moisture damage. The estimated relative change was determined using the negative binomial model estimate relative to the “no damage” category, indicated here as a dashed line at a value of 1.

The strongest associations seen for group 1 ERMI fungi were for door trim swab samples. In these samples, group 1 fungi were consistently higher in visibly damaged rooms, with some relative changes in abundances larger than 3.0 and all *P* values being ≤0.01 (Fig. 3, Table S5), although increases were not consistently monotonic for damage variables with more than two groups. Similar but weaker associations were seen for damage assessed at the home level (Fig. 3, Table S5). In contrast, group 1 fungi in door trim swab samples collected 6 months after building damage assessment showed strongly decreased relative abundances in mold-damaged rooms, with relative differences in relative abundances as low as 0.1 and *P* values of ≤0.01 (Table S5).

For vacuum dust and dust fall collector samples, no associations of group 1 fungi with contemporaneous damage were apparent (Table S6). For these sample types, group 1 fungi measured 6 months after building assessment also showed few consistent associations with damage indicators, with the exception that in later dust fall collector sampling, group 1 fungi had significantly increased relative abundances in homes with mold and other damage (but not those with visible mold alone). With the group 1 fungi, the relative changes and the *P* values in the statistical models were fairly consistent between taxonomic assignments with global and reference singletons (Tables S5 and S6). The limited patterns observed for relative abundances of group 1 fungi in vacuum samples and dust fall collector samples were not observed when examining the absolute abundance (data not shown).

### Approach 3: ecological distance between indoor and outdoor communities.

The comparison of an indoor sample to an outdoor sample is one method for assessing building damage recommended by the American Conference of Governmental and Industrial Hygienists (16). This is based on the expectation that, without an indoor source of growth, the fungi indoors should represent a proportional but filtered representation of the outdoor community (23). We hypothesized that the ecological distance of indoor communities to outdoor-air communities would be greater in water-damaged homes.

**FIG 4:**
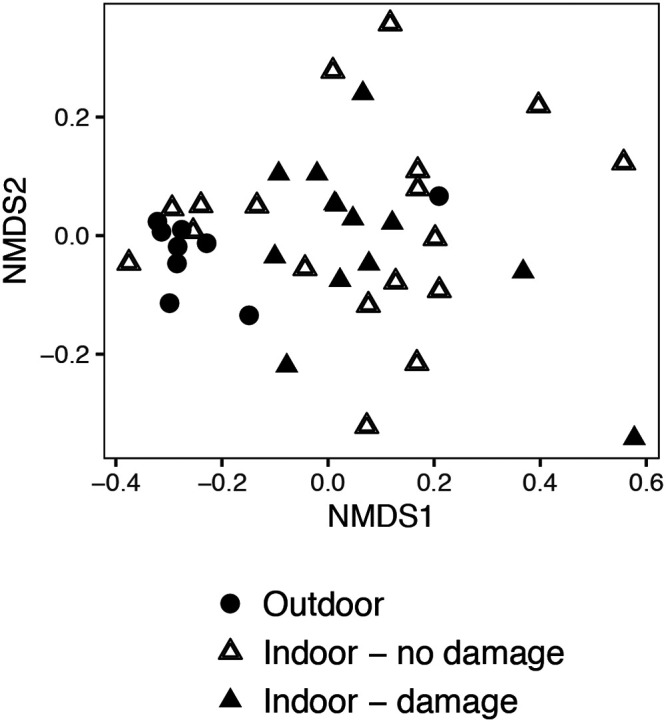
Example of a nonmetric multidimensional scaling (NMDS) plot of the ecological distances between the fungi in outdoor and indoor samples. Shown here are the relationships between the fungal compositions of outdoor dust fall collector samples and indoor dust fall collector samples in homes with and without visible mold damage, in Brooklyn, in the winter sampling period.

The Bray-Curtis ecological distances between fungal communities identified inside homes by various sampling types and those in outdoor settled dust were compared across homes with different levels of assessed damage. For these comparisons, including those between indoor and outdoor settled dust, the ecological distances generally were not greater in those homes with observed damage (Table S7). Figure 4 shows a representative example of one of these comparisons between outdoor and indoor samples across homes with and without damage. Ecological distances between indoor and outdoor samples were less in the summer than in the winter, and within a season, less between indoor and outdoor settled dust (collected by the same method, dust fall collectors) than between other indoor sample types and outdoor settled dust. Retaining only those taxa with a relative abundance greater than 0.1% decreased the ecological distances between indoor and outdoor samples, but those distances were not greater in homes with observed damage (data not shown). Furthermore, the number of shared taxa between indoor and outdoor samples was not decreased in those homes with damage compared to homes with no observed damage (data not shown).

### Approach 4: identifying indicator taxa.

Because water damage alters fungal communities in homes, we hypothesized that statistical tools could identify particular taxa that are present at increased concentrations in damp homes due to growth on damp materials. Analyses to identify indicator taxa found several associations between individual taxa and water damage status for vacuum samples (*n* = 14 indicator taxa) and dust fall collector samples (*n* = 21 taxa) (both shown in Table 2), as well as door trim swab samples (*n* = 107 taxa) (Table S8). The indicator taxon methods of ANCOM and LefSe identified taxa associated with mold damage (yes/no), while TITAN linked the change in abundance of a taxon to a particular gradient; in this case, the gradient was the surface area of mold damage. Overall, the strengths of the associations were weak, as reflected by relatively small effect sizes for the individual statistics. In vacuum samples, the yeasts Knufia epidermidis and Rhodotorula mucilaginosa were increased with water damage using all three methods of indicator taxa analyses. In door trim swab samples analyzed at the level of the home, all three analytical methods identified four indicators: the filamentous fungi Aspergillus unguis, Cladosporium halotolerans, Cyberlindnera jadinii, and Toxicocladosporium irritans. In fact, most of the taxa flagged by indicator taxa in door trim swab samples were filamentous fungi, such as species in the genera *Aspergillus*, *Penicillium*, and *Wallemia*. For all indicator taxon analysis of door trim swab samples at the room level, associations were weak.

**TABLE 2:** Indicator taxon analysis conducted on vacuum and dust fall collector samples^*a*^

Sampling method, taxonomy	Taxon ID^*b*^	ANCOM (detection level)	LEfSe (effect size)	TITAN	
				*Z* score	Change point (m^2^)
Vacuum					
Candida tropicalis	ASV_39		3.8	3.1	0.37
Coniosporium apollinis	ASV_314		3.2		
Curvularia inaequalis	ASV_488		3.1		
Dipodascaceae species	ASV_431		2.1		
Epicoccum brasiliense	ASV_270		2.6	6.9	2.65
Fusarium species	ASV_86	0.7	3.6		
Herpotrichiellaceae species	ASV_9300		2.0		
Holtermanniella wattica	ASV_1688		2.1		
Kazachstania humilis	ASV_1120		2.2	5.4	0.70
Knufia epidermidis	ASV_46	0.6	4.0	3.7	2.04
Papiliotrema laurentii	ASV_187			4.2	0.42
Rhodotorula mucilaginosa	ASV_61	0.8	3.8	4.0	0.42
Saccharomycetales species	ASV_519	0.7		4.9	0
Trichomeriaceae species	ASV_521	0.6	2.3		

Dust fall collectors					
Aspergillus insuetus	ASV_127		3.5	7.2	3.02
Aspergillus pseudodeflectus	ASV_67		3.5	6.8	2.74
Aspergillus puniceus	ASV_344		2.9		
Aspergillus ruber	ASV_80		3.4		
Aspergillus species	ASV_673		2.4		
Aspergillus versicolor	ASV_637		2.1		
Aspergillus versicolor	ASV_208			6.3	2.74
Cladosporium halotolerans	ASV_36			3.6	3.02
Exidia saccharina	ASV_1589		2.2		
Naganishia albida	ASV_60		3.9		
Naganishia diffluens	ASV_63	0.7	3.8		
Neocucurbitaria species	ASV_1985		2.4		
Penicillium citrinum	ASV_66		3.3		
Peniophora incarnata	ASV_255			5.8	1.16
Peniophora species	ASV_282		2.4		
Rhodotorula mucilaginosa	ASV_8		3.7		
Sistotrema oblongisporum	ASV_772		2.3		
Stereum complicatum	ASV_315		2.9		
Symmetrospora foliicola	ASV_514		2.7		
Talaromyces species	ASV_253		2.6		
Wallemia species	ASV_98		2.7		

a Only the species with significant associations (*P* < 0.05) are included. Reported values are the response strengths that are the output of the individual analysis.

b ID, identification number.

Of the 139 ASVs considered indicators by any of the three methods, only 9 were included in the targeted analyses: Aspergillus penicillioides, A. ruber, A. tamarii, A. unguis, A. versicolor, Cladosporium sphaerospermum, Epicoccum nigrum, Penicillium citrinum, and Rhodotorula mucilaginosa were in the group with known a_w_ requirements (Data Set S1), and A. penicillioides, A. unguis, A. versicolor, and C. sphaerospermum were also in ERMI group 1 (Data Set S2).

Overall taxonomic richness (observed, Shannon diversity, and inverse Simpson index) did not differ between homes with and without visible mold damage for any of the sample types used, nor for any building damage indicator (data not shown). Among sample types, vacuum dust samples displayed a greater richness of fungi than dust fall collector samples or swab samples of door trims.

## DISCUSSION

With the goal of developing a quantifiable fungal signature of moisture damage in buildings, we applied several strategies to a survey of homes. These strategies included a novel approach that identified particular fungi considered relevant to damp indoor environments due to either previously determined moisture requirements for growth or previously reported increased abundance in damp homes. This targeted approach draws on the power of high-throughput, amplicon-based sequencing to characterize the broad community while allowing the identification of specific groups of fungi in looking for a signal of moisture damage amid the noise that comes from the rich microbiological diversity in environmental samples. We then compared this new approach to more commonly applied approaches that look for signals of mold damage by considering the entire mycobiome.

In general, targeted approaches correlated more closely with estimates of building water damage than did whole-mycobiome methods, although the correlations were not consistent across the specific fungal groups and sampling methods. For example, in vacuum samples, hydrophilic fungi had higher relative abundances in water-damaged homes, but mesophilic fungi had unexpectedly lower relative abundances in homes with visible mold and xerophilic fungi showed no trends. Furthermore, in door trim dust sampled from water-damaged rooms, it was xerophilic fungi and ERMI group 1 fungi (themselves mostly xerophilic) that had higher relative abundances.

These inconsistencies could have multiple causes: for example, varied effects of different levels and sources of building moisture on fungal biology, the differing targets of specific environmental sampling methods, or technical artifacts of microbiome sequencing. Regarding biology, different types of water intrusion could create different moisture conditions in damaged building materials that could influence the fungi that proliferate. For example, a plumbing leak might create a wet region bordered by circles of decreasingly wet regions, and the relative amount of each region would influence the proportions of xerophiles, mesophiles, and hydrophiles growing on the damaged surfaces. In contrast to a plumbing leak, periods of higher relative humidity (RH) might only briefly and sparingly wet building materials, favoring the growth of xerophiles. Regarding sampling, floor vacuum samples, settled airborne dust on dust fall collectors, and door trim swab samples recover fungi deposited over different time periods. In our data, stronger microbial associations with damage were seen with vacuum floor dust and door trim swab samples than with dust fall collector samples, suggesting that longer-integrated dust samples provided stronger signals from moisture damage. Regarding analyses, because there is a limit to the number of amplified DNA segments that can be sequenced per sample with amplicon-based technology, an abundance of one fungal type depresses the number of DNA sequence reads of other fungi in that sample (24). Thus, in comparing several samples from one home, one sample may seem to have, relative to other samples, an increase in one specific fungal type and a decrease in others when in fact, although that type of fungus has increased in one sample, the other types are actually in equal abundance in all samples.

While further work is needed to clarify the relationships between the types of building damage, the specific fungal signatures, and the types of environmental dust collected, these results indicate that targeted approaches examining particular groups within a house mycobiome may have more power to detect the fungal signature of moisture damage across different homes than current untargeted approaches.

### Deciding which fungi to target and how to analyze them.

The biggest challenge with the targeted approach is identifying which taxa to include. Here, we selected taxa in two ways: taxa with reported minimum moisture requirements for growth and taxa reported to be in greater abundance in damaged homes. However, other possible approaches include choosing fungi based on their ability to initiate growth after desiccation (25) or to grow on damp building materials (e.g., see references 26 and 27). Also, how a_w_ thresholds are chosen to categorize fungal moisture requirements can affect analyses. Here, we defined three groups using a_w_ classifications and terminology similar to those of Park et al. (28), but others have defined four a_w_ groups (18). Repeating our targeted approach 1 with these four groups, however, produced results similar to those using three groups (data not shown). Additionally, fungal response to a specific a_w_ also involves the dimension of time, which was generally not considered in our sources of data on fungal moisture requirements. For example, Johansson et al. (29) found no growth on building materials at 75% RH after 12 weeks, but growth began at 16 weeks and beyond. Understanding the impact of time on minimum moisture requirements of fungal species is highly relevant for predicting fungal growth in buildings over the months and years over which elevated moisture events can occur.

Other challenges concern the current sequence-based identification of fungi known to inhabit water-damaged homes, many of which were initially recognized from cultivation. About one-third of the species for which we collected a_w_ requirements for growth have had a name change (Data Set S1 in the supplemental material). Another concern is that the gene region used to identify the fungi (the internal transcribed spacer 1 [ITS1] region) recognizes genera and species groups, but not necessarily individual species (30). Approximately 20% of the sequences we obtained could not be taxonomically resolved to species and were disregarded in our targeted approaches. An additional concern is that reference databases are not exhaustive, and they vary in their treatment of singleton species. In our data, despite the larger number of unresolved taxa when taxonomy was assigned with the UNITE database with global singletons, this database provided stronger relationships between fungal groups and building damage, suggesting that a data set with a larger taxonomic scope, even if more likely to contain taxonomic errors, better identifies differential patterns in relative abundance. Despite these uncertainties in species-based identification of fungi using modern sequencing technology, uncertainties which are not unique to the field of indoor air, fungal ecologists have made efforts similar to the targeted approaches used here. The program FunGuild assigns lifestyle and functional information to fungal taxa identified from high-throughput sequencing in order to study ecological guilds (31). A similar annotation tool could be developed to include features important to fungi in the built environment.

The particular features of a damp building that are most strongly associated with a microbial response are not known. We considered many metrics summarizing observed water damage and mold, including visible mold, other damage, moisture content of building materials measured through a moisture meter, and window condensation. We hypothesized these to be important damage indicators, although other metrics—singly or combined—may be better linked to fungal signatures. Among the metrics considered here, simple indicators based on visible mold were most consistently linked to the microbiological data. We saw the strongest relationships between building damage and microbial responses when they were near each other, physically and temporally. Thus, we expect that the better aligned the microbial measurements are to any water damage, in both space and time, the better the ability to detect the damage.

Finally, we explored differences in the abundances of microbial groups, using both relative abundance and absolute abundance, across home damage levels. Absolute abundance could only be determined for vacuum and dust fall collector samples, because their standardized collection strategy allowed meaningful comparisons of biomass. The concentration of an environmental agent is considered to provide a less meaningful estimate of exposure than the load per area of that agent. In sequencing analysis, the relative abundance of a species as a proportion of the total community is analogous to the concentration in a sample, and absolute abundance is analogous to load. Therefore, the absolute abundance of particular fungi should be more informative than their relative abundance because, in theory, it adjusts for variation in total biomass (32). Thus, we expected building damage to be more strongly related to absolute microbial abundance than to relative abundance but saw only limited support for this. In dust fall collector samples, the relative abundances of hydrophilic fungi showed a tendency to increase with damage. While absolute abundances, in contrast, showed large and significant increases, this was apparently an artifact from a single home. This strengthened relationship for absolute abundance was not seen across other groups of fungi and environmental-sample types. Still, given the theoretical advantage of absolute abundance, we consider it a promising metric for a quantitative signal of moisture damage.

### Untargeted approaches.

While certain differences between indoor and outdoor microbial communities seem to be logical indicators of an indoor source of fungal growth, this approach has received little scientific investigation. A key exception is a study by Miller et al. (9), who reported that the proportions of indoor aerosol samples from a home that differed significantly in fungal rankings from pooled outdoor samples were significantly and positively related to the total area of visible or hidden mold growth in the home, determined by opening all walls in the home. Recently, Hegarty et al. (8) found that compositional similarity to outdoor samples decreased with increasing proximity of indoor sampling locations to visible mold in the basement of the building.

This indoor-outdoor comparison approach has many advantages. It allows for regional variation in what species comprise the “normal fungal ecology” (33). Also, with amplicon sequencing, taxonomic identifications of individual sequences are not considered when calculating ecological distance or percentages of shared taxa between two samples. In our data set, we observed no patterns of association between outdoor-indoor ecological distances and dampness/mold, possibly because of limited numbers of outdoor samples and the performance of most comparisons across collection methods.

While sequence-based approaches and the breadth of microbial diversity they reveal seem promising for identifying taxa that indicate specific conditions, our study highlights some current limitations. For indoor environments, it is not clear whether the apparent indicator species identified are actually growing on building materials. The indicator taxon analysis of door trim swab samples here flagged Aspergillus unguis, Cladosporium halotolerans, and Toxicocladosporium irritans, all known from indoor environments (18, 34, 35). Yet indicator taxon analysis also flagged Cyberlindnera jadinii, a yeast used in the feed industry as a protein supplement whose natural habitat is not known (36). Because chance associations between some species and particular conditions are likely in any one study, such findings are best considered hypotheses for future exploration.

### Conclusion.

This study applied multiple old and new approaches for identifying quantitative microbial signals of damp, moldy buildings, using amplicon data that are inherently semiqualitative (37). Within the habitat of buildings, we found that (i) both fungi selected by known moisture requirements and fungi reported to occur at elevated levels in water-damaged buildings were elevated in certain sample types in damp or moldy buildings, (ii) among time-integrated dust samples, samples with longer but unspecified time-integration periods showed stronger relationships with moisture conditions than samples of shorter but defined time-integration periods, and (iii) despite issues of taxonomic identification and resolution in amplicon data sets, targeted approaches using the current annotation tools showed stronger relationships with the relevant environmental parameter (building dampness) than untargeted approaches. Thus, we suggest that a targeted approach using specific preselected fungi has potential for producing a quantitative microbial indicator of moisture failure in buildings. Toward that goal, we would recommend two next steps. One, the process of selecting the fungal taxa to target needs further development, including additional input from the research community focused on indoor mycology and taxonomy. Two, the patterns seen in our novel analyses could be further explored in existing data sets from other building populations. Moisture damage is likely to leave a detectable signal in the microbiome of a building, and further research to identify that signal is important. Broadly, our results indicate that isolating particular species from the amplicon-sequenced mycobiome produces a greatly reduced but potentially powerful data set for detecting subtle ecological patterns.

## MATERIALS AND METHODS

### Building selection and recruitment process.

The data analyzed here were collected between January and September 2015, in a study in New York City, NY, USA. We partnered with two local community organizations, The Red Hook Initiative and Community Voices Heard, to recruit volunteer households in Brooklyn and Manhattan through physical flyers, electronic messaging, and announcements posted at the organization offices. We recruited participants in both public housing and privately owned buildings in the same boroughs. The first step of recruitment involved a screening questionnaire asking potential volunteers whether they suffered any presence or history of dampness problems or any water damage from the recent Hurricane Sandy (29 October 2012). Based on the screening, participants were grouped initially into water damage and no water damage groups but kept blind to this grouping. Participants were not excluded based on age, gender, or the number of tenants occupying dwellings, but a maximum of two units per building were included.

Each participant was informed about the purpose of the study, the nature of the measurements to be taken inside their home, and information to be collected at the beginning of each of two measurement periods. Additionally, each participant was informed about confidentiality of data collected during the study and received financial compensation at the end of each measurement period. This study was approved by the University of California Committee for the Protection of Human Subjects under protocol 2014-08-6589.

### Building inspection and sample collection.

Study data were collected in two sets of 3-week measurements. Each set of measurements was completed within a 3-month period, the first period beginning in January 2015 (winter samples) and the second in August 2015 (summer samples). During the initial visit in each home, information about the building and dwelling was obtained through occupant-completed survey questionnaires and researcher-collected observations and measurements. In the occupant surveys, participants were asked to report the following: any signs of dampness inside the unit; the presence, type, and frequency of use of any heating or cooling system in the dwelling, including window-mounted air-conditioning units; the presence of portable air cleaners; and the presence of humidifiers and dehumidifiers. Participants were also asked about the number of occupants in their home (including pets), and the occupants’ cooking and cleaning habits.

Also during the initial visit, the researchers recorded observations of any water damage and other signs of increased dampness, including visible mold, water maps, cracks, and paint or material peeling off walls. The room, location (wall, ceiling, etc.), and size of each damage area were recorded. Indicators of inadequate ventilation, such as water condensation on windows, were additionally recorded. Real-time data on living room (LR) indoor air temperature (TLR, °C), indoor air relative humidity (RHLR, %), and CO_2_ concentrations (ppm) were collected in study dwellings using a TIM10 data logger, with 6-min sampling intervals. In the sleep area of the master bedroom (BR) or second bedroom, real-time data on indoor air temperature (TBR, °C) and indoor air relative humidity (RHBR, %) were collected using Hobo data loggers (U12-100), also with 6-min sampling intervals. Additionally, real-time use of the heating or cooling systems and of window opening in each home was captured with an additional data logger (EL USB-1; Lascar Electronics).

Suspended dust collection samplers were left in homes during the initial visit to collect passively settled airborne dust over the sampling period. Samplers were open, empty, sterile, 10-cm-diameter petri dishes with no growth medium, as previously described (23), placed in living rooms or bedrooms. Heights of samplers varied between 1 and 2 m from the floor. We refer to this environmental sample type as “dust fall collector samples.” Where possible and in compliance with New York City Housing Authority (NYCHA) rental policies, we also deployed passive airborne dust collectors outdoors, modified to prevent the entry of precipitation and placed on top of air-conditioning units, balconies, or roofs.

After a 3-week period, researchers returned to the homes. In addition to retrieving the dust fall collectors, additional measurements and environmental samples were taken. The moisture content in exterior-adjacent walls (MC, %) was determined with a noninvasive pinless moisture meter (MO-290; Extech). For each home, these standardized moisture measurements were taken in the kitchen, living room, bathroom, and bedroom. One measurement was taken per room, only on an exterior wall, at a center point oriented horizontally between windows and vertically between the floor and ceiling. Moisture measurement readings are used to indicate relatively elevated moisture content in the walls. We considered readings above 15 to be elevated. Additionally, swabs (Floq swabs; Copan Diagnostics) were run along the top surface of the upper trim of selected doors to provide integrated microbiological samples of settled dust (38). Swab samples were taken in at least two trim locations in each home: the bathroom door opening to the hallway and the front door opening to the home exit. For some homes, additional door trim swab samples were collected from bedrooms opening to a hallway. Finally, floor dust was vacuumed from a 1-m^2^ area in the living room for a period of 1 min, using a Dustream collector (Indoor Biotechnologies) attached to a vacuum wand (SupraQuik portable canister vacuum; Riccar). Indoor samples were frozen within 8 h of collection and stored at −20°C until processed.

In the second sampling period, approximately 6 months after the first sampling period, researchers returned to many of the same homes to collect temperature, RH, and CO_2_ concentration data again, as well as to take additional dust samples (dust fall collectors, door trim swabs, and vacuumed dust). This period included no other researcher-collected observations or measurements and no occupant-completed questionnaires. To replace initially participating homes that did not participate in the second sampling period, additional residential units were recruited and included in the second measurement period. For these new homes, researchers collected dust samples and occupant-completed questionnaires. However, because these summer-only homes had no researcher-collected observations or measurements related to mold or water damage, they are not included in the current analyses.

### Defining damage indicators.

Because there is no established standard practice for assessing building moisture damage, we explored various potential indicators. The eight building damage indicators that we used and their possible response levels are shown in Table 3, as are the approximate number of homes in each category (the precise number varied with the environmental sample type). All were used in analyses that considered damage in the entire home, and four were used in analyses of room-specific damage. Three indicators were based solely on the presence and size (wall area) of visible mold. An additional three indicators also included other forms of damage potentially caused by excess moisture: “other damage” included cracking paint, paint and other materials coming off the wall, and water maps. One indicator reflected high levels of measured moisture. A whole-home composite damage index (range, 0 to 6) was defined as the sum of four other indicators: number of separate mold areas (0, 1, or 2+), size of total mold damage area (categorized as 0, 1, or 2, representing none, low, or high), whether any moisture meter readings exceeded 15 (0 or 1, representing no/yes), and any observed window condensation with microbial growth (0 or 1, representing no/yes).

**TABLE 3:** Building damage indicators and possible levels for each of the indicators used in this study

Building damage indicator	House-level analysis		Room-level analysis	
	Categories	Approx no. in each category^*a*^	Categories	Approx no. in each category^*a*^
Mold damage	No, yes	15, 16	No, yes	69, 9
No. of mold areas	0, 1, 2+	15, 12, 4		
Mold size (m^2^ of surface area)	0, 0 < *x* < 1.9, *x* ≥ 1.9	15, 10, 6	0, 0 < *x* < 0.9, *x* ≥ 0.9	69, 5, 4
Mold and other damage^*b*^	No, yes	5, 26	No, yes	59, 19
No. of mold and other damage areas	0, 1, 2+	5, 17, 9		
Mold and other damage size (m^2^ of surface area)	0, 0 < *x* < 1.9, *x* ≥ 1.9	5, 19, 7	0, 0 < *x* < 0.9, *x* ≥ 0.9	59, 11, 8
No. of moisture meter readings >15	0, 1+	20, 11		
Composite damage index	0, 1–2, 3–6	11, 11, 9		

a The precise number of samples in each category varied with the environmental sample type. For house-level categories, numbers of vacuum samples are shown, and for room-level categories, numbers of door trim swab samples are shown.

b “Other damage” includes cracking paint, paint and other materials coming off the wall, and water maps.

### Fungal community assessment.

Experimental samplers and control samplers (sterile samplers that had not been deployed) were treated identically in all laboratory manipulations. For each sample, genomic DNA (gDNA) was extracted using a phenol-chloroform-isoamyl alcohol extraction protocol with Qiagen DNeasy PowerSoil HTP 96 kits, as previously described (23). The ITS1 region, which is a segment of the rRNA gene that acts as a barcode of fungal identity (39), was amplified and sequenced using Illumina MiSeq 250 paired-end sequencing, following methods previously described (40).

Bioinformatic steps are included as File S1 in the supplemental material. Briefly, we used only the R1 reads due to low quality in the R2 reads, and the ITS1 region was extracted using isxpress (41). Sequences were then processed into amplicon sequence variants (ASVs) in DADA2 (42) in the R environment (43). ASVs infer the genetic sequences that would have been present in the sample before processing (overcoming potential errors in amplification and sequencing) and are considered to show greater taxonomic resolution than operational taxonomic units (OTUs) (44). The ASVs were assigned taxonomic classification using the UNITE fungal reference database, version 02.02.2019 (19). Because of the methods used to assign a taxonomic name to an ASV, the same sequence can be assigned different names depending on the reference database used to infer taxonomy. Also, different ASVs can be assigned the same taxonomic name, even when using a single reference database. Contaminant sequences were identified using the decontam package (45) with a prevalence threshold of 50% of the value for the negative-control samples and removed from the data set. Data processing relied heavily on the phyloseq package (46) in R.

Quantitative PCR (qPCR) was performed on the genomic extracts from vacuum and dust fall collector samples. These two sample types were implemented in a way that standardizes the collection of environmental material. Door trim swabs, in contrast, can vary in the collection of environmental material if swabbed over different lengths of building material or if different pressures are used while swabbing. The qPCR assays were conducted to determine fungal biomass using ITS primers FF2/FR1 (47) and SYBR green. The quantification standard consisted of plasmids that had been constructed in-house from amplification with Aspergillus fumigatus genomic extract. Reactions were done on a Bio-Rad CFX96 Touch real-time PCR detection system.

### Analytical approaches.

For approaches 1, 2, and 3, fungal communities were evaluated at the different levels of each damage indicator (Table 3). For home-level comparisons, in those homes with at least two door trim swabs (range, *n* = 2 to 4), the door trim swab results were averaged to derive home-level door trim data for comparison with home-level damage indicators. For room-level comparisons, room-specific door trim swab samples were compared with room-specific damage indicators. For approach 4, analysis looked for indicator taxa associated with particular damage indicators depending on the particular indicator taxon method.

### Approach 1.

We first generated a list of fungal species with previously determined minimum a_w_ values. Starting with those fungal taxa included in Table 1 in Flannigan and Miller (18) and checking the primary sources cited in that table, we identified the original and any additional data in the primary sources. Using additional identified references, we increased the number of fungal taxa with a reported minimum a_w_ required for growth (48–56). In this data collection, we targeted references focused on indoor environments, to prioritize taxa that were important in fungal growth on building materials, but did not aim for an exhaustive list of all fungal species with known minimum moisture requirements (many of which have been investigated due to their growth on foods). Data Set S1 provides the species included, current taxonomic names, and references used.

For each taxon, we recorded the minimum a_w_ value (or equilibrium relative humidity [RH], where water activity = RH/100) for which growth was observed, on any growth substrate, in the 23 to 27°C temperature range. If a fungal taxon was identified in more than one citation, the overall minimum a_w_ value reported for that taxon was calculated, as well as the mean of the minimum values from each citation. For each species’ moisture requirement, we used the mean of the minimum a_w_ values reported. Current taxonomy was determined using Index Fungorum (http://www.indexfungorum.org/). In some instances, species names have changed. In other instances, previously separate species were combined into one species, named using the current taxonomy. The curated list used here contains information for 108 fungal species, including 23 *Aspergillus* species, 18 *Penicillium* species, and 15 *Cladosporium* species. We note that we consider these updated taxonomic assignments preliminary, and more authoritative taxonomic identification may be necessary.

Following taxonomic identifications in the UNITE databases either with global or reference singletons, we produced, for approach 1, two reduced data sets containing only the species of interest (i.e., out of the 108) that were also identifiable in the UNITE databases. Bioinformatic processing steps for forming subsets of particular taxa from the full community table, in order to produce the reduced data sets, are shown in File S1.

For comparison of microbial levels in two or more groups (e.g., more or less damaged homes), an analysis method was needed that was appropriate for the typical distribution of microbial-count data. The microbiome data we observed (Fig. S1) were neither normal nor log-normal, and the variance of taxon counts typically exceeded the mean (i.e., data were overdispersed). To analyze these data for abundance differences of fungi across different levels of assessed building damage, we used negative binomial regression models (57). Negative binomial models were chosen because they fit the microbial count data better than Poisson models (often used for count data), based on the Akaike information criterion (AIC) (data not shown). Note that comparisons of individual fungal taxa, whose counts include very high proportions of zeros, likely require “zero-adjusted” negative binomials. However, because our analyses of summed groups of taxa contained few zero counts, unadjusted negative binomial models sufficed. Modeling used the glm.nb function in R on the counts of fungal groups, offset by the log of the total number of reads in the sample. The offset in the model allows comparison of relative abundance, rather than absolute counts, between groups (57). Negative binomial model coefficients represent the differences in mean log relative abundances between samples; that is, the exponentiated model coefficients represent the multiplicative change in the relative abundances in the damaged group(s) compared to the reference group. For prediction variables with two categories (e.g., present versus absent), *P* values were determined using the *Z* test statistic on the model coefficient. For prediction variables with three categories (e.g., none, low, or high), *P* values were determined using a two-degrees-of-freedom chi-square test on the full and reduced models, where full models had both terms of the covariate while reduced models had neither. We ran models on both of the reduced data sets generated by taxonomic identification with global and reference singleton species.

We also compared the absolute abundances of fungi across levels of assessed building damage. The relative abundance of a taxon (a proportion) is converted to absolute abundance (a load) by multiplying the relative abundance by the total fungal concentration (i.e., taxon relative abundance in a sample multiplied by the total fungal biomass in that sample, as determined with qPCR) (58). This transformation allows estimation of environmental loads, theoretically more relevant for assessing both building damage and health-related exposures. Note that absolute abundance could be examined only in vacuum and dust fall collector samples, but not swab samples, because the former two had standardized collection areas and therefore were appropriate for qPCR. As described above, negative binomial regression models were used to test for differences in the abundances of fungal groups, although no offset was used in modeling absolute abundance.

### Approach 2.

As with the fungi with known moisture requirements for growth in approach 1, negative binomial linear models were run to test for differences in relative abundance across different levels of assessed damage in approach 2. Again, taxonomic identification was performed separately using both reduced data sets and generated with reference or global singleton species. Also, the absolute abundances of group 1 fungi were examined in vacuum and dust fall collector samples.

### Approach 3.

In approach 3, the ecological distance, using the Bray-Curtis index, was calculated in a pairwise fashion between indoor samples and outdoor samples. Four composite outdoor samples were generated by taking the mean of each taxon within each of the different location-time point pairings: Brooklyn-winter, *n* = 10; Brooklyn-summer, *n* = 21; Manhattan-winter, *n* = 7; and Manhattan-summer, *n* = 3. Each indoor sample in a given location (Brooklyn or Manhattan) at a given time point (winter or summer) was compared with the corresponding outdoor pooled sample (9). Because outdoor-community samples were taken solely with dust fall collectors, most of the comparisons between indoor and outdoor communities were made across different sample types. That is, settled dust was compared indoors to outdoors, but we also separately compared fungal communities in indoor door trim swab samples with communities in outdoor dust fall collector samples. Statistical differences between the distribution of these ecological distances were determined using Wilcoxon-Mann-Whitney tests for two-category building damage variables or Kruskal-Wallis tests for three-category variables.

### Approach 4.

In approach 4, we used various statistical approaches to identify taxa associated with damage. First, we used ANCOM (59) and identified all taxa that were differentially abundant, under a loose cutoff of 0.6, in homes with and without mold damage. Second, we used LEfSe (60), which is based on linear discriminant analysis (LDA), to identify taxa associated with mold (yes/no), using a value of 2 as the threshold of the logarithmic LDA score. Finally, we used TITAN, which identifies taxa associated with environmental gradients (61), using the size of visible mold, either in the room or the entire housing unit, as the environmental gradient. For the TITAN analysis, we included only ASVs observed at least 3 times in 10% of the sites.

Separately, in order to compare richness (α diversity) across sample types and building conditions, we estimated the observed richness, the Shannon diversity, and the inverse Simpson index in homes with and without mold damage. Diversity metrics were compared using *t* tests.

### Data availability.

Raw amplicon data were deposited at the NCBI Sequence Read Archive with BioProject accession number PRJNA603120.

## Supplementary Material

Supplemental file 1

Supplemental file 2

Supplemental file 3
