# Supplementary material for: Fungal Signature of Moisture Damage in Buildings: Identification by Targeted and Untargeted Approaches with Mycobiome Data
Source: Appl Environ Microbiol. 2020 Aug 18;86(17):e01047-20. doi: 10.1128/AEM.01047-20 (PMC7440782; doi:10.1128/AEM.01047-20)
Supplement: Supplemental file 1 [file AEM.01047-20-s0003.pdf]

Identifying a fungal signature of moisture damage in buildings by taking a targeted approach with microbiome data

Rachel I. Adams, Iman Sylvain, Michal P. Spilak, John W. Taylor, Michael S. Waring, and Mark J. Mendell

Supplementary Files

## Table of Contents

***Figure S1 Distribution of count data*** ........ ***2***

***Table S1 Hydrophilic, mesophilic, and xerophilic fungi in vacuum and dustfall collectors*** ...... ***3***

***Table S2 Hydrophilic, mesophilic, and xerophilic fungi in door trim swabs*** ........ ***5***

***Table S3 Taxonomy assignments using UNITE fungal reference database with reference singletons*** ........ ***7***

***Table S4 Absolute abundance of fungal groups*** ........ ***9***

***Table S5 ERMI Group 1 fungi in door trim swabs*** ........ ***11***

***Table S6 ERMI Group 1 fungi in vacuum dust and dustfall collectors*** ........ ***13***

***Table S7 Ecological distances between indoor and outdoor samples*** ........ ***16***

***Table S8 Indicator taxa analysis conducted on fungi in door trip swabs*** ........ ***17***

***Text S1 Supplementary Text*** ........ ***20***

**Comparison of UNITE global singletons versus UNITE reference singletons** ........ **20**

**Bioinformatic scripts in R: Sequence processing of amplicon data** ........ **21**

**Bioinformatic scripts in R: Isolating taxa of interest from a phyloseq object** ........ **31**

# Figure S1 Distribution of count data

Sum of hydrophilic, mesophilic, xerophilic, and ERMI Group 1 fungi in study samples, ranked from highest to lowest sum.

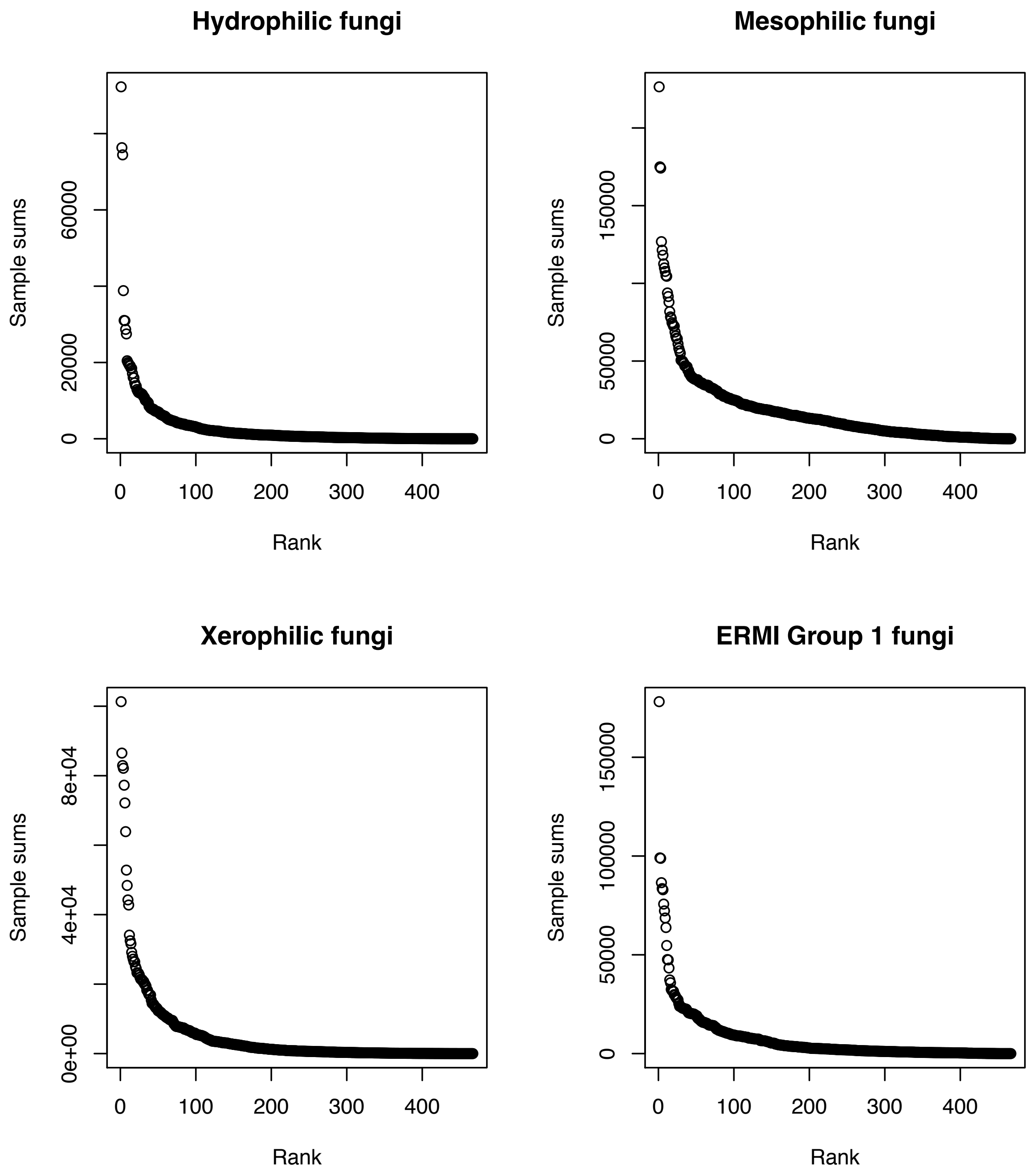

## Table S1 Hydrophilic, mesophilic, and xerophilic fungi in vacuum and dustfall collectors

Relative abundance of hydrophilic, mesophilic, and xerophilic fungi in vacuum samples and dustfall collectors for homes with and without building damage; means of the relative abundance in each group and estimated relative change from a negative binomial model, with p-values of the model coefficients.

| | Vacuum Samples | | | Dustfall Collectors | | |
|---|---|---|---|---|---|---|
| | | Negative binomial model | | | Negative binomial model | |
| Building damage category | Group means of the relative abundance | Estimated relative change[1] | p-value[2] | Group means of the relative abundance | Estimated relative change | p-value |
| **Hydrophilic fungi** | | | | | | |
| Visible mold damage (yes/no) | 0.020, 0.048 | 1.0, 2.4 | **0.01** | 0.020, 0.034 | 1.0, 1.6 | 0.27 |
| # Mold areas (None, low, high) | 0.020, 0.047, 0.053[3] | 1.0, 2.3, 2.6[3] | **0.04** | 0.020, 0.042, 0.018 | 1.0, 2.1, 0.9 | 0.30 |
| Mold size ($m^2$) (None, low, high) | 0.020, 0.048, 0.048 | 1.0, 2.4, 2.4 | **0.04** | 0.020, 0.045, 0.025 | 1.0, 2.2, 1.2 | 0.41 |
| Composite damage index | 0.024, 0.039, 0.043 | 1.0, 1.7, 1.8 | 0.33 | 0.018, 0.039, 0.025 | 1.0, 2.2, 1.4 | 0.33 |
| Mold and other damage (yes/no) | 0.016, 0.038 | 1.0, 2.4 | 0.06 | 0.011, 0.032 | 1.0, 2.8 | **0.02** |
| # Mold and other damage areas | 0.016, 0.034, 0.046 | 1.0, 2.1, 2.8 | 0.18 | 0.011, 0.038, 0.020 | 1.0, 3.4, 1.8 | **0.05** |
| Mold and other damage size ($m^2$) | 0.016, 0.036, 0.043 | 1.0, 2.3, 2.7 | 0.22 | 0.011, 0.036, 0.027 | 1.0, 3.2, 2.3 | 0.07 |
| # Moisture meter readings >15 | 0.039, 0.028 | 1.0, 0.7 | 0.35 | 0.023, 0.028 | 1.0, 1.2 | 0.72 |
| **Mesophilic fungi** | | | | | | |
| Visible mold damage (yes/no) | 0.055, 0.023 | 1.0, 0.4 | **<0.01** | 0.130, 0.137 | 1.0, 1.1 | 0.85 |
| # Mold areas (None, low, high) | 0.055, 0.023, 0.024[3] | 1.0, 0.4, 0.4[3] | **0.02** | 0.130, 0.151, 0.113 | 1.0, 1.2, 0.9 | 0.82 |
| Mold size ($m^2$) (None, low, high) | 0.055, 0.024, 0.021 | 1.0, 0.4, 0.4 | **0.02** | 0.130, 0.179, 0.106 | 1.0, 1.4, 0.8 | 0.50 |
| Composite damage index | 0.059, 0.027, 0.027 | 1.0, 0.4, 0.5 | **0.03** | 0.109, 0.195, 0.122 | 1.0, 1.8, 1.1 | 0.15 |
| Mold and other damage (yes/no) | 0.058, 0.034 | 1.0, 0.6 | 0.18 | 0.140, 0.128 | 1.0, 0.9 | 0.74 |
| # Mold and other damage areas | 0.058, 0.042, 0.020 | 1.0, 0.7, 0.3 | **0.05** | 0.140, 0.135, 0.115 | 1.0, 1, 0.8 | 0.84 |
| Mold and other damage size ($m^2$) | 0.058, 0.039, 0.022 | 1.0, 0.7, 0.4 | 0.14 | 0.140, 0.143, 0.108 | 1.0, 1, 0.8 | 0.66 |
| # Moisture meter readings >15 | 0.042, 0.033 | 1.0, 0.8 | 0.47 | 0.126, 0.146 | 1.0, 1.2 | 0.59 |
| **Xerophilic fungi** | | | | | | |

| | | | | | | |
|---|---|---|---|---|---|---|
| Visible mold damage (yes/no) | 0.012, 0.017 | 1.0, 1.4 | 0.38 | 0.090, 0.112 | 1.0, 1.2 | 0.56 |
| # Mold areas (None, low, high) | 0.012, 0.018, 0.016[3] | 1.0, 1.4, 1.3[3] | 0.68 | 0.090, 0.124, 0.090 | 1.0, 1.4, 1 | 0.75 |
| Mold size ($m^2$) (None, low, high) | 0.012, 0.020, 0.013 | 1.0, 1.6, 1.1 | 0.54 | 0.090, 0.073, 0.141 | 1.0, 0.8, 1.6 | 0.49 |
| Composite damage index | 0.014, 0.010, 0.020 | 1.0, 0.7, 1.4 | 0.43 | 0.100, 0.064, 0.121 | 1.0, 0.6, 1.2 | 0.46 |
| Mold and other damage (yes/no) | 0.017, 0.014 | 1.0, 0.8 | 0.67 | 0.110, 0.090 | 1.0, 0.8 | 0.59 |
| # Mold and other damage areas | 0.017, 0.015, 0.011 | 1.0, 0.9, 0.7 | 0.73 | 0.110, 0.071, 0.127 | 1.0, 0.6, 1.2 | 0.38 |
| Mold and other damage size ($m^2$) | 0.017, 0.015, 0.013 | 1.0, 0.8, 0.7 | 0.87 | 0.110, 0.067, 0.123 | 1.0, 0.6, 1.1 | 0.33 |
| # Moisture meter readings >15 | 0.016, 0.012 | 1.0, 0.7 | 0.47 | 0.094, 0.102 | 1.0, 1.1 | 0.83 |

[1] Exponential of the negative binomial model estimate, interpreted as the estimated relative change in the relative abundance

[2] For prediction variables with two categories (e.g. present vs. absent), the p-values were determined using the z test statistic on the model coefficient. For prediction variables with three categories (e.g., none, low, high), p-values were determined using a two degree-of-freedom chi-square test on the full and reduced models.

[3] High damage category has fewer than 5 individuals in that group

[4] Bold, p-value ≤ 0.05

## Table S2 Hydrophilic, mesophilic, and xerophilic fungi in door trim swabs

Relative abundance of the hydrophilic, mesophilic, and xerophilic fungi in door trim swabs where damage assessment was made within a room or combined for a home-level assessment; means of the relative abundance in each group and estimated relative change from a negative binomial model, with p-values of the model coefficients. Building damage assessment that occurred simultaneously with dust collected are referred to as "winter", while dust collected that occurred six months following building damage assessment are labelled "summer."

| **Hydrophilic fungi** | | | | | | |
|---|---|---|---|---|---|---|
| | Room-level | | | House-level | | |
| | | Negative binomial model | | | Negative binomial model | |
| Building damage category | Group means of the relative abundance | Estimated relative change[1] | p-value[2] | Group means of the relative abundance | Estimated relative change | p-value |
| *Winter* | | | | | | |
| Mold damage (y/n) | 0.046, 0.032 | 1.0, 0.7 | 0.51 | 0.039, 0.037 | 1.0, 0.9 | 0.88 |
| Mold size | 0.046, 0.040, 0.023[3] | 1.0, 0.9, 0.5[3] | 0.71 | 0.039, 0.043, 0.026[3] | 1.0, 1.1, 0.7[3] | 0.73 |
| Any damage (y/n) | 0.044, 0.045 | 1.0, 1.0 | 0.98 | 0.032, 0.043 | 1.0, 1.4 | 0.35 |
| Any damage size | 0.044, 0.036, 0.057 | 1.0, 0.8, 1.3 | 0.81 | 0.032, 0.043, 0.042 | 1.0, 1.4, 1.3 | 0.65 |
| | | | | | | |
| *Summer* | | | | | | |
| Mold damage (y/n) | 0.023, 0.076 | 1.0, 3.3 | 0.12 | 0.029, 0.025 | 1.0, 0.9 | 0.75 |
| Mold size | 0.023, 0.104, 0.057[3] | 1.0, 4.5, 2.5[3] | 0.20 | 0.029, 0.012, 0.042 | 1.0, 0.4, 1.5 | 0.25 |
| Any damage (y/n) | 0.023, 0.049 | 1.0, 2.1 | 0.21 | 0.032, 0.025 | 1.0, 0.8 | 0.60 |
| Any damage size | 0.023, 0.054, 0.043 | 1.0, 2.3, 1.9 | 0.42 | 0.032, 0.020, 0.035 | 1.0, 0.6, 1.1 | 0.57 |
| **Mesophilic fungi** | | | | | | |
| | Room-level | | | House-level | | |
| | | Negative binomial model | | | Negative binomial model | |
| Building damage category | Group means of the relative abundance | Estimated relative change | p-value | Group means of the relative abundance | Estimated relative change | p-value |
| *Winter* | | | | | | |
| Mold damage (y/n) | 0.189, 0.272 | 1.0, 1.4 | 0.24 | 0.198, 0.176 | 1.0, 0.9 | 0.62 |
| Mold size | 0.189, 0.270, 0.274[3] | 1.0, 1.4, 1.5[3] | 0.47 | 0.198, 0.129, 0.259[3] | 1.0, 0.7, 1.3[3] | 0.19 |
| Any damage (y/n) | 0.189, 0.228 | 1.0, 1.2 | 0.41 | 0.207, 0.182 | 1.0, 0.9 | 0.54 |
| Any damage size | 0.189, 0.223, 0.237 | 1.0, 1.2, 1.3 | 0.70 | 0.207, 0.175, 0.203 | 1.0, 0.8, 1 | 0.74 |
| | | | | | | |

| | | | | | | |
|---|---|---|---|---|---|---|
| *Summer* | | | | | | |
| Mold damage (y/n) | 0.206, 0.243 | 1.0, 1.2 | 0.65 | 0.173, 0.264 | 1.0, 1.5 | **0.04** |
| Mold size | 0.206, 0.242, 0.243[3] | 1.0, 1.2, 1.2[3] | 0.9 | 0.173, 0.272, 0.251 | 1.0, 1.6, 1.5 | 0.12 |
| Any damage (y/n) | 0.231, 0.167 | 1.0, 0.7 | 0.25 | 0.198, 0.216 | 1.0, 1.1 | 0.71 |
| Any damage size | 0.231, 0.145, 0.200 | 1.0, 0.6, 0.9 | 0.42 | 0.198, 0.203, 0.246 | 1.0, 1, 1.2 | 0.73 |
| **Xerophilic fungi** | | | | | | |
| | Room-level | | | House-level | | |
| | | Negative binomial model | | | Negative binomial model | |
| Building damage category | Group means of the relative abundance | Estimated relative change | p-value | Group means of the relative abundance | Estimated relative change | p-value |
| *Winter* | | | | | | |
| Mold damage (y/n) | 0.046, 0.131 | 1.0, 2.9 | **0.03** | 0.061, 0.086 | 1.0, 1.4 | 0.28 |
| Mold size | 0.046, 0.196, 0.050[3] | 1.0, 4.3, 1.1[3] | **0.02** | 0.061, 0.079, 0.099[3] | 1.0, 1.3, 1.6[3] | 0.50 |
| Any damage (y/n) | 0.042, 0.098 | 1.0, 2.3 | **0.02** | 0.056, 0.075 | 1.0, 1.3 | 0.31 |
| Any damage size | 0.042, 0.111, 0.080 | 1.0, 2.7, 1.9 | **0.04** | 0.056, 0.075, 0.075 | 1.0, 1.3, 1.3 | 0.61 |
| | | | | | | |
| *Summer* | | | | | | |
| Mold damage (y/n) | 0.068, 0.004 | 1.0, 0.1 | **<0.01** | 0.070, 0.039 | 1.0, 0.6 | 0.16 |
| Mold size | 0.068, 0.003, 0.005[3] | 1.0, 0.0, 0.1[3] | **0.01** | 0.070, 0.048, 0.025 | 1.0, 0.7, 0.4 | 0.25 |
| Any damage (y/n) | 0.069, 0.034 | 1.0, 0.5 | 0.20 | 0.041, 0.064 | 1.0, 1.5 | 0.34 |
| Any damage size | 0.069, 0.018, 0.058 | 1.0, 0.3, 0.8 | 0.22 | 0.041, 0.078, 0.030 | 1.0, 1.9, 0.7 | 0.18 |

[1] Exponential of the negative binomial model estimate, interpreted as the estimated relative change in the relative abundance

[2] For prediction variables with two categories (e.g. present vs. absent), the p-values were determined using the z test statistic on the model coefficient. For prediction variables with three categories (e.g., none, low, high), p-values were determined using a two degree-of-freedom chi-square test on the full and reduced models.

[3] High damage category has fewer than 5 individuals in that group

[4] Bold, p-value ≤ 0.05

## Table S3 Taxonomy assignments using UNITE fungal reference database with reference singletons

Relative abundance of hydrophilic, mesophilic, and xerophilic fungi, with taxonomy assigned using the UNITE fungal reference database with reference singletons, in vacuum samples and dustfall collectors for homes with and without building damage; means of the relative abundance in each group and estimated relative change from a negative binomial model, with p-values of the model coefficients.

| | Vacuum Samples | | | Dustfall Collectors | | |
|---|---|---|---|---|---|---|
| | | Negative binomial model | | | Negative binomial model | |
| Building damage category | Group means of the relative abundance | Estimated relative change[1] | p-value[2] | Group means of the relative abundance | Estimated relative change | p-value |
| **Hydrophilic fungi** | | | | | | |
| Visible mold damage (yes/no) | 0.021, 0.043 | 1.0, 2.1 | **0.03** | 0.020, 0.034 | 1.0, 1.7 | 0.25 |
| # Mold areas (None, low, high) | 0.021, 0.041, 0.047[3] | 1.0, 2.0, 2.2[3] | 0.12 | 0.020, 0.044, 0.015 | 1.0, 2.2, 0.8 | 0.23 |
| Mold size ($m^2$) (None, low, high) | 0.021, 0.042, 0.043 | 1.0, 2.0, 2.1 | 0.12 | 0.020, 0.044, 0.026 | 1.0, 2.3, 1.3 | 0.38 |
| Composite damage index | 0.024, 0.034, 0.039 | 1.0, 1.4, 1.6 | 0.54 | 0.018, 0.038, 0.024 | 1.0, 2.2, 1.4 | 0.34 |
| Mold and other damage (yes/no) | 0.016, 0.035 | 1.0, 2.2 | 0.08 | 0.010, 0.032 | 1.0, 3.4 | **<0.01** |
| # Mold and other damage areas | 0.016, 0.031, 0.042 | 1.0, 2, 2.7 | 0.22 | 0.010, 0.039, 0.018 | 1.0, 4.1, 1.9 | **0.02** |
| Mold and other damage size ($m^2$) | 0.016, 0.034, 0.039 | 1.0, 2.2, 2.5 | 0.28 | 0.010, 0.037, 0.026 | 1.0, 3.9, 2.7 | **0.03** |
| # Moisture meter readings >15 | 0.036, 0.025 | 1.0, 0.7 | 0.31 | 0.023, 0.027 | 1.0, 1.2 | 0.74 |
| **Mesophilic fungi** | | | | | | |
| Visible mold damage (yes/no) | 0.066, 0.043 | 1.0, 0.7 | 0.17 | 0.169, 0.189 | 1.0, 1.1 | 0.61 |
| # Mold areas (None, low, high) | 0.066, 0.043, 0.045[3] | 1.0, 0.6, 0.7[3] | 0.40 | 0.169, 0.211, 0.149 | 1.0, 1.3, 0.9 | 0.58 |
| Mold size ($m^2$) (None, low, high) | 0.066, 0.047, 0.038 | 1.0, 0.7, 0.6 | 0.36 | 0.169, 0.198, 0.182 | 1.0, 1.2, 1.1 | 0.85 |
| Composite damage index | 0.072, 0.049, 0.039 | 1.0, 0.7, 0.5 | 0.27 | 0.148, 0.226, 0.182 | 1.0, 1.5, 1.2 | 0.23 |
| Mold and other damage (yes/no) | 0.076, 0.050 | 1.0, 0.7 | 0.30 | 0.195, 0.165 | 1.0, 0.8 | 0.43 |
| # Mold and other damage areas | 0.076, 0.059, 0.033 | 1.0, 0.8, 0.4 | 0.17 | 0.195, 0.176, 0.142 | 1.0, 0.9, 0.7 | 0.54 |

| | | | | | | |
|---|---|---|---|---|---|---|
| Mold and other damage size ($m^2$) | 0.076, 0.055, 0.038 | 1.0, 0.7, 0.5 | 0.36 | 0.195, 0.163, 0.167 | 1.0, 0.8, 0.9 | 0.73 |
| # Moisture meter readings >15 | 0.060, 0.045 | 1.0, 0.7 | 0.36 | 0.166, 0.195 | 1.0, 1.2 | 0.46 |
| **Xerophilic fungi** | | | | | | |
| Visible mold damage (yes/no) | 0.013, 0.017 | 1.0, 1.3 | 0.44 | 0.091, 0.112 | 1.0, 1.2 | 0.58 |
| # Mold areas (None, low, high) | 0.013, 0.018, 0.016[3] | 1.0, 1.4, 1.3[3] | 0.73 | 0.091, 0.124, 0.090 | 1.0, 1.4, 1 | 0.75 |
| Mold size ($m^2$) (None, low, high) | 0.013, 0.020, 0.013 | 1.0, 1.5, 1 | 0.57 | 0.091, 0.073, 0.142 | 1.0, 0.8, 1.6 | 0.47 |
| Composite damage index | 0.015, 0.011, 0.020 | 1.0, 0.7, 1.3 | 0.41 | 0.102, 0.065, 0.121 | 1.0, 0.6, 1.2 | 0.43 |
| Mold and other damage (yes/no) | 0.017, 0.014 | 1.0, 0.8 | 0.69 | 0.110, 0.092 | 1.0, 0.8 | 0.61 |
| # Mold and other damage areas | 0.017, 0.016, 0.011 | 1.0, 0.9, 0.6 | 0.69 | 0.110, 0.073, 0.129 | 1.0, 0.7, 1.2 | 0.39 |
| Mold and other damage size ($m^2$) | 0.017, 0.015, 0.013 | 1.0, 0.9, 0.7 | 0.86 | 0.110, 0.069, 0.124 | 1.0, 0.6, 1.1 | 0.35 |
| # Moisture meter readings >15 | 0.017, 0.012 | 1.0, 0.7 | 0.41 | 0.096, 0.103 | 1.0, 1.1 | 0.85 |

[1] Exponential of the negative binomial model estimate, interpreted as the estimated relative change in the relative abundance

[2] For prediction variables with two categories (e.g. present vs. absent), the p-values were determined using the z test statistic on the model coefficient. For prediction variables with three categories (e.g., none, low, high), p-values were determined using a two degree-of-freedom chi-square test on the full and reduced models.

[3] High damage category has fewer than 5 individuals in that group

[4] Bold, p-value ≤ 0.05

# Table S4 Absolute abundance of fungal groups

The absolute abundance of hydrophilic, mesophilic, and xerophilic fungi in vacuum and dustfall collectors for homes with and without building damage; means of the absolute abundance in each group, intepreted as gene copy equivalements, and estimated relative change from a negative binomial model, with p-values of the model coefficients.

| | Vacuum Samples | | | Dustfall Collectors | | |
|---|---|---|---|---|---|---|
| | | Negative binomial model | | | Negative binomial model | |
| Building damage category | Group means of the absolute abundance | Estimated relative change[1] | p-value[2] | Group means of the absolute abundance | Estimated relative change | p-value |
| **Hydrophilic fungi** | | | | | | |
| Visible mold damage (yes/no) | 1652, 1079 | 1.0, 0.7 | 0.35 | 4, 24 | 1.0, 5.9 | **0.01** |
| # Mold areas (None, low, high) | 1652, 747, 1994[3] | 1.0, 0.5, 1.2[3] | 0.22 | 4, 32, 1[3] | 1.0, 7.8, 0.2[3] | **0.01** |
| Mold size ($m^2$) (None, low, high) | 1652, 780, 1529 | 1.0, 0.5, 0.9 | 0.37 | 4, 12, 37 | 1.0, 2.9, 9 | **0.03** |
| Composite damage index | 1814, 933, 1298 | 1.0, 0.5, 0.7 | 0.48 | 5, 8, 25 | 1.0, 1.6, 5.1 | 0.15 |
| Mold and other damage (yes/no) | 2830, 1073 | 1.0, 0.4 | 0.10 | 6, 13 | 1.0, 2 | 0.36 |
| # Mold and other damage areas | 2830, 747, 1653 | 1.0, 0.3, 0.6 | 0.05 | 6, 17, 2 | 1.0, 2.7, 0.3 | 0.14 |
| Mold and other damage size ($m^2$) | 2830, 752, 1897 | 1.0, 0.3, 0.7 | **0.04** | 6, 5, 25 | 1.0, 0.9, 3.9 | 0.18 |
| # Moisture meter readings >15 | 1335, 1419 | 1.0, 1.1 | 0.90 | 6, 19 | 1.0, 2.9 | 0.17 |
| **Mesophilic fungi** | | | | | | |
| Visible mold damage (yes/no) | 5568, 1525 | 1.0, 0.3 | **0.01** | 173, 57 | 1.0, 0.3 | 0.20 |
| # Mold areas (None, low, high) | 5568, 1410, 1870[3] | 1.0, 0.3, 0.3[3] | **0.05** | 173, 75, 2[3] | 1.0, 0.4, 0[3] | 0.16 |
| Mold size ($m^2$) (None, low, high) | 5568, 1636, 1341 | 1.0, 0.3, 0.2 | **0.05** | 173, 43, 71 | 1.0, 0.2, 0.4 | 0.48 |
| Composite damage index | 6435, 1771, 1866 | 1.0, 0.3, 0.3 | 0.06 | 230, 34, 53 | 1.0, 0.1, 0.2 | 0.13 |
| Mold and other damage (yes/no) | 7194, 2705 | 1.0, 0.4 | 0.14 | 318, 30 | 1.0, 0.1 | **<0.01** |
| # Mold and other damage areas | 7194, 3153, 1859 | 1.0, 0.4, 0.3 | 0.20 | 318, 31, 28 | 1.0, 0.1, 0.1 | **0.02** |
| Mold and other damage size ($m^2$) | 7194, 2869, 2260 | 1.0, 0.4, 0.3 | 0.27 | 318, 18, 50 | 1.0, 0.1, 0.2 | **0.01** |

| | | | | | | |
|---|---|---|---|---|---|---|
| # Moisture meter readings >15 | 4053, 2579 | 1.0, 0.6 | 0.42 | 182, 39 | 1.0, 0.2 | 0.07 |
| **Xerophilic fungi** | | | | | | |
| Visible mold damage (yes/no) | 862, 2076 | 1.0, 2.4 | 0.13 | 36, 91 | 1.0, 2.5 | 0.28 |
| # Mold areas (None, low, high) | 862, 2500, 804[3] | 1.0, 2.9, 0.9[3] | 0.19 | 36, 119, 7[3] | 1.0, 3.3, 0.2[3] | 0.22 |
| Mold size ($m^2$) (None, low, high) | 862, 2978, 573 | 1.0, 3.5, 0.7 | 0.07 | 36, 17, 165 | 1.0, 0.5, 4.6 | 0.17 |
| Composite damage index | 904, 2583, 861 | 1.0, 2.9, 1 | 0.20 | 45, 16, 113 | 1.0, 0.4, 2.5 | 0.24 |
| Mold and other damage (yes/no) | 873, 1606 | 1.0, 1.8 | 0.42 | 64, 47 | 1.0, 0.7 | 0.70 |
| # Mold and other damage areas | 873, 2090, 694 | 1.0, 2.4, 0.8 | 0.24 | 64, 29, 91 | 1.0, 0.4, 1.4 | 0.51 |
| Mold and other damage size ($m^2$) | 873, 1885, 851 | 1.0, 2.2, 1 | 0.45 | 64, 8, 112 | 1.0, 0.1, 1.7 | **0.03** |
| # Moisture meter readings >15 | 1745, 943 | 1.0, 0.5 | 0.32 | 39, 84 | 1.0, 2.1 | 0.38 |

[1] Exponential of the negative binomial model estimate, interpreted as the estimated relative change in the relative abundance

[2] For prediction variables with two categories (e.g. present vs. absent), the p-values were determined using the z test statistic on the model coefficient. For prediction variables with three categories (e.g., none, low, high), p-values were determined using a two degree-of-freedom chi-square test on the full and reduced models.

[3] High damage category has fewer than 5 individuals in that group

[4] Bold, p-value ≤ 0.05

# Table S5 ERMI Group 1 fungi in door trim swabs

Supplementary Table 8: Relative abundance of ERMI Group 1 fungi in door trim in rooms homes with and without building damage in comparison to homes with and without building damage; means of the relative abundance in each group and estimated relative change from a negative binomial model, with p-values of the model coefficients. Comparison of the sampling that occurred contemporaneously (“winter”) and following the building damage assessment (“summer”) as well as with taxonomy assignment using the UNITE fungal reference database with global and reference singletons.

| **With Global Singletons** | | | | | | |
|---|---|---|---|---|---|---|
| | Room-level | | | Home-level | | |
| | | Negative binomial model | | | Negative binomial model | |
| Building damage category | Group means of the relative abundance | Estimated relative change[1] | p-value[2] | Group means of the relative abundance | Estimated relative change | p-value |
| *Winter* | | | | | | |
| Mold damage (y/n) | 0.079, 0.247 | 1.0, 3.1 | **<0.01** | 0.087, 0.140 | 1.0, 1.6 | **0.05** |
| Mold size | 0.079, 0.280, 0.206[3] | 1.0, 3.6, 2.6[3] | **<0.01** | 0.087, 0.115, 0.183[3] | 1.0, 1.3, 2.1[3] | 0.08 |
| Any damage (y/n) | 0.074, 0.174 | 1.0, 2.4 | **<0.01** | 0.079, 0.115 | 1.0, 1.4 | 0.10 |
| Any damage size | 0.074, 0.174, 0.173 | 1.0, 2.4, 2.3 | **0.01** | 0.079, 0.107, 0.140 | 1.0, 1.3, 1.8 | 0.20 |
| | | | | | | |
| *Summer* | | | | | | |
| Mold damage (y/n) | 0.088, 0.007 | 1.0, 0.1 | **<0.01** | 0.093, 0.065 | 1.0, 0.7 | 0.31 |
| Mold size | 0.088, 0.007, 0.008[3] | 1.0, 0.1, 0.1[3] | **<0.01** | 0.093, 0.079, 0.045 | 1.0, 0.8, 0.5 | 0.37 |
| Any damage (y/n) | 0.084, 0.055 | 1.0, 0.7 | 0.35 | 0.061, 0.090 | 1.0, 1.5 | 0.30 |
| Any damage size | 0.084, 0.027, 0.099 | 1.0, 0.3, 1.2 | 0.14 | 0.061, 0.107, 0.050 | 1.0, 1.8, 0.8 | 0.17 |
| **With Reference Singletons** | | | | | | |
| | Room-level | | | Home-level | | |
| Building damage category | Group means of the relative abundance | Estimated relative change[1] | p[2] | Group means of the relative abundance | Estimated relative change | p |
| *Winter* | | | | | | |
| Mold damage (y/n) | 0.111, 0.241 | 1.0, 2.2 | **0.05** | 0.122, 0.166 | 1.0, 1.4 | 0.21 |
| Mold size | 0.111, 0.284, 0.187[3] | 1.0, 2.5, 1.7[3] | 0.08 | 0.122, 0.129, 0.232[3] | 1.0, 1.1, 1.9[3] | 0.17 |
| Any damage (y/n) | 0.107, 0.187 | 1.0, 1.7 | **0.05** | 0.124, 0.141 | 1.0, 1.1 | 0.56 |
| Any damage size | 0.107, 0.193, 0.178 | 1.0, 1.8, 1.7 | 0.13 | 0.124, 0.131, 0.174 | 1.0, 1.1, 1.4 | 0.57 |
| | | | | | | |
| *Summer* | | | | | | |
| Mold damage (y/n) | 0.105, 0.015 | 1.0, 0.1 | **<0.01** | 0.114, 0.097 | 1.0, 0.8 | 0.60 |

| | | | | | | |
|---|---|---|---|---|---|---|
| Mold size | 0.105, 0.026, 0.008[3] | 1.0, 0.2, 0.1[3] | **0.01** | 0.114, 0.112, 0.075 | 1.0, 1, 0.7 | 0.63 |
| Any damage (y/n) | 0.102, 0.069 | 1.0, 0.7 | 0.37 | 0.079, 0.120 | 1.0, 1.5 | 0.22 |
| Any damage size | 0.102, 0.036, 0.117 | 1.0, 0.4, 1.2 | 0.19 | 0.079, 0.134, 0.085 | 1.0, 1.7, 1.1 | 0.27 |

[1] Exponential of the negative binomial model estimate, interpreted as the estimated relative change in the relative abundance

[2] For prediction variables with two categories (e.g. present vs. absent), the p-values were determined using the z test statistic on the model coefficient. For prediction variables with three categories (e.g., none, low, high), p-values were determined using a two degree-of-freedom chi-square test on the full and reduced models.

[3] High damage category has fewer than 5 individuals in that group

[4] Bold, p-value ≤ 0.05

# Table S6 ERMI Group 1 fungi in vacuum dust and dustfall collectors

Relative abundance of ERMI Group 1 fungi in vacuum samples and dustfall collectors in homes with and without building damage; means of the relative abundance in each group and estimated relative change from a negative binomial model, with p-values of the model coefficients. Comparison of the sampling that occurred contemporaneously ("winter") and following the building damage assessment ("summer") as well as with taxonomy assignment using the UNITE fungal reference database with global and reference singletons.

| *Winter* | | | | | | |
|---|---|---|---|---|---|---|
| Taxonomy with global singletons | | | | | | |
| | Vacuum Samples | | | Dustfall Collectors | | |
| | | Negative binomial model | | | Negative binomial model | |
| Building damage category | Group means of the relative abundance | Estimated relative change[1] | p-value[2] | Group means of the relative abundance | Estimated relative change* | p-value |
| Visible mold damage (yes/no) | 0.020, 0.025 | 1.0, 1.2 | 0.48 | 0.112, 0.138 | 1.0, 1.2 | 0.45 |
| # Mold areas (None, low, high) | 0.020, 0.025, 0.025[3] | 1.0, 1.2, 1.2[3] | 0.78 | 0.112, 0.152, 0.113 | 1.0, 1.4, 1 | 0.62 |
| Mold size ($m^2$) (None, low, high) | 0.020, 0.028, 0.020 | 1.0, 1.4, 1 | 0.56 | 0.112, 0.101, 0.166 | 1.0, 0.9, 1.5 | 0.42 |
| Composite damage index | 0.020, 0.021, 0.029 | 1.0, 1.1, 1.5 | 0.55 | 0.122, 0.087, 0.148 | 1.0, 0.7, 1.2 | 0.37 |
| Mold and other damage (yes/no) | 0.022, 0.023 | 1.0, 1 | 0.96 | 0.134, 0.113 | 1.0, 0.8 | 0.54 |
| # Mold and other damage areas | 0.022, 0.025, 0.019 | 1.0, 1.1, 0.8 | 0.71 | 0.134, 0.096, 0.147 | 1.0, 0.7, 1.1 | 0.37 |
| Mold and other damage size ($m^2$) | 0.022, 0.024, 0.019 | 1.0, 1.1, 0.9 | 0.85 | 0.134, 0.090, 0.146 | 1.0, 0.7, 1.1 | 0.28 |
| # Moisture meter readings >15 | 0.022, 0.025 | 1.0, 1.1 | 0.69 | 0.114, 0.133 | 1.0, 1.2 | 0.57 |
| Taxonomy with reference singletons | | | | | | |
| | Vacuum Samples | | | Dustfall Collectors | | |
| Building damage category | Group means of the relative abundance | Estimated relative change | p-value | Group means of the relative abundance | Estimated relative change | p-value |
| Visible mold damage (yes/no) | 0.025, 0.037 | 1.0, 1.5 | 0.21 | 0.135, 0.179 | 1.0, 1.3 | 0.32 |
| # Mold areas (None, low, high) | 0.025, 0.041, 0.027[3] | 1.0, 1.6, 1.1[3] | 0.34 | 0.135, 0.203, 0.137 | 1.0, 1.5, 1 | 0.43 |
| Mold size ($m^2$) (None, low, high) | 0.025, 0.046, 0.022 | 1.0, 1.9, 0.9 | 0.13 | 0.135, 0.112, 0.230 | 1.0, 0.8, 1.7 | 0.18 |

| | | | | | | |
|---|---|---|---|---|---|---|
| Composite damage index | 0.025, 0.038, 0.031 | 1.0, 1.5, 1.2 | 0.57 | 0.152, 0.096, 0.196 | 1.0, 0.6, 1.3 | 0.16 |
| Mold and other damage (yes/no) | 0.030, 0.031 | 1.0, 1.1 | 0.90 | 0.173, 0.137 | 1.0, 0.8 | 0.41 |
| # Mold and other damage areas | 0.030, 0.037, 0.020 | 1.0, 1.3, 0.7 | 0.25 | 0.173, 0.124, 0.164 | 1.0, 0.7, 0.9 | 0.51 |
| Mold and other damage size ($m^2$) | 0.030, 0.035, 0.021 | 1.0, 1.2, 0.7 | 0.48 | 0.173, 0.098, 0.194 | 1.0, 0.6, 1.1 | 0.08 |
| # Moisture meter readings >15 | 0.034, 0.026 | 1.0, 0.8 | 0.48 | 0.139, 0.172 | 1.0, 1.2 | 0.45 |
| | | | | | | |
| *Summer* | | | | | | |
| Taxonomy with global singletons | | | | | | |
| | Vacuum Samples | | | Dustfall Collectors | | |
| Building damage category | Group means of the relative abundance | Estimated relative change | p-value | Group means of the relative abundance | Estimated relative change | p-value |
| Visible mold damage (yes/no) | 0.028, 0.028 | 1.0, 1 | 1.00 | 0.063, 0.053 | 1.0, 0.8 | 0.66 |
| # Mold areas (None, low, high) | 0.028, 0.030, 0.018[3] | 1.0, 1.1, 0.6[3] | 0.79 | 0.063, 0.057, 0.009[3] | 1.0, 0.9, 0.1[3] | 0.44 |
| Mold size ($m^2$) (None, low, high) | 0.028, 0.036, 0.015 | 1.0, 1.3, 0.5 | 0.25 | 0.063, 0.054, 0.052 | 1.0, 0.9, 0.8 | 0.91 |
| Composite damage index | 0.030, 0.021, 0.035 | 1.0, 0.7, 1.2 | 0.43 | 0.073, 0.049, 0.045 | 1.0, 0.7, 0.6 | 0.50 |
| Mold and other damage (yes/no) | 0.025, 0.030 | 1.0, 1.2 | 0.57 | 0.031, 0.074 | 1.0, 2.4 | 0.02 |
| # Mold and other damage areas | 0.025, 0.032, 0.021 | 1.0, 1.3, 0.9 | 0.59 | 0.031, 0.083, 0.038 | 1.0, 2.6, 1.2 | 0.04 |
| Mold and other damage size ($m^2$) | 0.025, 0.034, 0.015 | 1.0, 1.4, 0.6 | 0.16 | 0.031, 0.082, 0.043 | 1.0, 2.6, 1.4 | 0.05 |
| # Moisture meter readings >15 | 0.031, 0.021 | 1.0, 0.7 | 0.26 | 0.066, 0.042 | 1.0, 0.6 | 0.34 |
| | | | | | | |
| Taxonomy without reference singletons | | | | | | |
| | Vacuum Samples | | | Dustfall Collectors | | |
| Building damage category | Group means of the relative abundance | Estimated relative change | p-value | Group means of the relative abundance | Estimated relative change | p-value |
| Visible mold damage (yes/no) | 0.036, 0.034 | 1.0, 0.9 | 0.83 | 0.076, 0.111 | 1.0, 1.5 | 0.35 |
| # Mold areas (None, low, high) | 0.036, 0.035, 0.027[3] | 1.0, 1, 0.7[3] | 0.92 | 0.076, 0.120, 0.011 | 1.0, 1.6, 0.1 | 0.22 |

| | | | | | | |
|---|---|---|---|---|---|---|
| Mold size ($m^2$) (None, low, high) | 0.036, 0.041, 0.022 | 1.0, 1.1, 0.6 | 0.52 | 0.076, 0.137, 0.059 | 1.0, 1.8, 0.8 | 0.34 |
| Composite damage index | 0.037, 0.028, 0.041 | 1.0, 0.7, 1.1 | 0.63 | 0.087, 0.108, 0.051 | 1.0, 1.2, 0.6 | 0.48 |
| Mold and other damage (yes/no) | 0.030, 0.037 | 1.0, 1.2 | 0.52 | 0.039, 0.109 | 1.0, 2.8 | 0.01 |
| # Mold and other damage areas | 0.030, 0.039, 0.029 | 1.0, 1.3, 1 | 0.66 | 0.039, 0.124, 0.044 | 1.0, 3.2, 1.1 | 0.01 |
| Mold and other damage size ($m^2$) | 0.030, 0.041, 0.024 | 1.0, 1.4, 0.8 | 0.40 | 0.039, 0.123, 0.049 | 1.0, 3.2, 1.3 | 0.02 |
| # Moisture meter readings >15 | 0.038, 0.028 | 1.0, 0.7 | 0.36 | 0.097, 0.046 | 1.0, 0.5 | 0.13 |

[1] Exponential of the negative binomial model estimate, interpreted as the estimated relative change in the relative abundance

[2] For prediction variables with two categories (e.g. present vs. absent), the p-values were determined using the z test statistic on the model coefficient. For prediction variables with three categories (e.g., none, low, high), p-values were determined using a two degree-of-freedom chi-square test on the full and reduced models.

[3] High damage category has fewer than 5 individuals in that group

[4] Bold, p-value ≤ 0.05

## Table S7 Ecological distances between indoor and outdoor samples

Ecological distances between indoor and outdoor samples in different environmental sample types for homes with different building damage

| | Vacuum | | Dustfall Collectors[1] | |
|---|---|---|---|---|
| **Winter** | | | | |
| Building damage category | Group means of the ecological distance | p-value[2] | Group means of the ecological distance | p-value |
| Visible mold damage (yes/no) | 0.83, 0.80 | 0.49 | 0.82, 0.87 | 0.36 |
| # Mold areas (None, low, high) | 0.83, 0.80, 0.82[3] | 0.52 | 0.82, 0.87, 0.86 | 0.62 |
| Mold size (m2) (None, low, high) | 0.83, 0.79, 0.83 | 0.24 | 0.82, 0.86, 0.87 | 0.65 |
| Composite damage index | 0.84, 0.8, 0.81 | 0.39 | 0.86, 0.74, 0.88 | **0.05** |
| Mold and other damage (yes/no) | 0.85, 0.81 | 0.37 | 0.80, 0.86 | 0.20 |
| # Mold and other damage areas | 0.85, 0.80, 0.81 | 0.60 | 0.80, 0.85, 0.87 | 0.41 |
| Mold and other damage size ($m^2$) | 0.85, 0.80, 0.83 | 0.28 | 0.80, 0.86, 0.85 | 0.40 |
| # Moisture meter readings >15 | 0.83, 0.79 | 0.56 | 0.84, 0.84 | 0.92 |
| | | | | |
| | Swabs - House level | | Swabs - Room level | |
| | | | | |
| | Group means of the ecological distance | p-value | Group means of the ecological distance | p-value |
| Visible mold damage (yes/no) | 0.82, 0.83 | 0.88 | 0.89, 0.87 | 0.38 |
| # Mold areas (None, low, high) | 0.82, 0.85, 0.73[3] | 0.28 | | |
| Mold size (m2) (None, low, high) | 0.82, 0.83, 0.82 | 0.96 | 0.89, 0.81, 0.94[3] | 0.22 |
| Composite damage index | 0.80, 0.84, 0.85 | 0.36 | | |
| Mold and other damage (yes/no) | 0.80, 0.84 | 0.29 | 0.89, 0.88 | 0.74 |
| # Mold and other damage areas | 0.80, 0.83, 0.85 | 0.50 | | |
| Mold and other damage size ($m^2$) | 0.80, 0.84, 0.85 | 0.54 | 0.89, 0.85, 0.93 | 0.40 |
| # Moisture meter readings >15 | 0.81, 0.84 | 0.52 | | |

1 Dustfall Collector column represents the only comparison of the same sampling method indoor and outdoor
2 Statistical differences determined using Wilcoxon-Mann-Whitney tests for two category building damage variables or Kruskal-Walllis tests for three-category variables
3 High damage category has fewer than 5 individuals in that group
4 Bold, p-value ≤ 0.05

## Table S8 Indicator taxa analysis conducted on fungi in door trip swabs

Indicator taxa analysis conducted on swab samples in the winter

| | | ANCOM | LEfSe | TITAN | |
|---|---|---|---|---|---|
| **Taxonomy** | **Taxon ID** | Detection level | Effect size | Z-score | Change point ($m^2$) |
| **Swabs - Home - Winter** | | | | | |
| Antrodiella onychoides | ASV_1975 | -- | 2.5 | -- | -- |
| Aspergillus penicillioides | ASV_813 | -- | 2.4 | -- | -- |
| Aspergillus tamarii | ASV_123 | -- | -- | 5.1 | 2.51 |
| Aspergillus unguis | ASV_47 | 0.7 | 3.6 | 6.8 | 3.62 |
| Aspergillus versicolor | ASV_1360 | -- | 2.1 | -- | -- |
| Byssomerulius corium | ASV_7348 | -- | 3.0 | -- | -- |
| Cabalodontia spp. | ASV_3329 | -- | 2.3 | -- | -- |
| Candida parapsilosis | ASV_10 | 0.7 | 4.0 | -- | -- |
| Ceraceomyces tessulatus | ASV_5469 | -- | 2.8 | -- | -- |
| Cladosporium halotolerans | ASV_36 | 0.9 | 4.1 | 4.4 | 0 |
| Cladosporium sphaerospermum | ASV_975 | -- | -- | 7.5 | 6.97 |
| Cladosporium spp. | ASV_68 | -- | 3.4 | 6.6 | 0.93 |
| Cryptococcus uniguttulatus | ASV_41 | -- | -- | 5.8 | 3.21 |
| Cyberlindnera jadinii | ASV_72 | 0.7 | 3.5 | 4.1 | 0.09 |
| Didymella glomerata | ASV_20 | 0.8 | 4.0 | -- | -- |
| Epicoccum nigrum | ASV_599 | -- | 2.0 | -- | -- |
| Helotiales spp. | ASV_4388 | -- | 2.5 | -- | -- |
| Microascus intricatus | ASV_1824 | -- | 2.4 | -- | -- |
| Microascus trigonosporus | ASV_3121 | -- | 2.1 | -- | -- |
| Naganishia albida | ASV_30 | 0.9 | 4.0 | -- | -- |
| Neocucurbitaria spp. | ASV_251 | -- | -- | 6.9 | 0.93 |
| Paracremonium spp. | ASV_4037 | -- | 2.4 | -- | -- |
| Paradendryphiella arenariae | ASV_792 | -- | 2.9 | -- | -- |
| Piloderma spp. | ASV_9240 | -- | 2.8 | -- | -- |
| Pleosporales spp. | ASV_5331 | -- | 2.6 | -- | -- |
| Sistotrema spp. | ASV_1202 | -- | 2.7 | -- | -- |
| Sordariales spp. | ASV_5134 | -- | 2.3 | -- | -- |
| Sporobolomyces roseus | ASV_2303 | -- | 3.2 | -- | -- |
| Sporobolomyces salicinus | ASV_1682 | -- | 2.0 | -- | -- |
| Stereum complicatum | ASV_1083 | -- | 2.1 | -- | -- |
| Stereum ostrea | ASV_2476 | -- | 2.8 | -- | -- |
| Stereum sanguinolentum | ASV_3790 | -- | 2.3 | -- | -- |

| | | | | | |
|---|---|---|---|---|---|
| Syncephalastrum spp. | ASV_9660 | -- | 2.1 | -- | -- |
| Talaromyces diversus | ASV_10132 | -- | 2.6 | -- | -- |
| Talaromyces minioluteus | ASV_6594 | -- | 2.9 | -- | -- |
| Toxicocladosporium irritans | ASV_19 | 0.7 | 4.1 | 6 | 3.21 |
| Toxicocladosporium irritans | ASV_843 | -- | 2.4 | -- | -- |
| Trechispora spp. | ASV_3053 | -- | 2.5 | -- | -- |
| Tremellomycetes spp. | ASV_592 | -- | 2.1 | -- | -- |
| Unidentified  fungus | ASV_3043 | -- | 2.0 | -- | -- |
| **Swabs - Room** | | | | | |
| Agaricomycetes spp. | ASV_562 | -- | 2.2 | -- | -- |
| Agaricomycetes spp. | ASV_3906 | -- | 2.4 | -- | -- |
| Agaricomycetes spp. | ASV_4263 | -- | 2.2 | -- | -- |
| Agaricomycetes spp. | ASV_4612 | -- | 2.2 | -- | -- |
| Amyloporia sinuosa | ASV_2374 | -- | 2.1 | -- | -- |
| Aspergillus pseudodeflectus | ASV_8882 | -- | 2.2 | -- | -- |
| Aspergillus unguis | ASV_47 | 0.6 | 3.8 | -- | -- |
| Aspergillus versicolor | ASV_1360 | -- | 2.8 | -- | -- |
| Aspergillus versicolor | ASV_7902 | -- | 2.3 | -- | -- |
| Cabalodontia subcretacea | ASV_5261 | -- | 2.1 | -- | -- |
| Candida metapsilosis | ASV_1560 | -- | 2.5 | -- | -- |
| Capnobotryella renispora | ASV_1480 | -- | 2.6 | -- | -- |
| Ceriporia spp. | ASV_5746 | -- | 2.0 | -- | -- |
| Cladosporium halotolerans | ASV_568 | -- | 2.1 | -- | -- |
| Cladosporium spp. | ASV_68 | -- | 2.9 | -- | -- |
| Cladosporium spp. | ASV_5770 | -- | 2.4 | -- | -- |
| Cladosporium spp. | ASV_5854 | -- | 2.6 | -- | -- |
| Cladosporium spp. | ASV_8104 | -- | 2.1 | -- | -- |
| Coniothyrium spp. | ASV_307 | -- | 2.6 | -- | -- |
| Cryptococcus uniguttulatus | ASV_41 | -- | 3.6 | -- | -- |
| Dothideales spp. | ASV_2820 | -- | 2.3 | -- | -- |
| Dothioraceae spp. | ASV_3425 | -- | 2.1 | -- | -- |
| Epicoccum nigrum | ASV_599 | -- | 2.8 | -- | -- |
| Eurotiales spp. | ASV_9642 | -- | 2.1 | -- | -- |
| Ganoderma spp. | ASV_711 | -- | 2.5 | -- | -- |
| Ganoderma spp. | ASV_899 | -- | 2.1 | -- | -- |
| Gelatoporia subvermispora | ASV_345 | -- | 2.2 | -- | -- |
| Grifola frondosa | ASV_4802 | -- | 2.6 | -- | -- |
| Hannaella spp. | ASV_1066 | -- | 2.6 | -- | -- |
| Heterochaete shearii | ASV_1952 | -- | 2.2 | -- | -- |

| | | | | | |
|---|---|---|---|---|---|
| Hypoxylon submonticulosum | ASV_2349 | -- | 2.4 | -- | -- |
| Jaapia argillacea | ASV_2643 | -- | 2.3 | -- | -- |
| Limonomyces spp. | ASV_1891 | -- | 2.6 | -- | -- |
| Madurella spp. | ASV_167 | -- | 2.9 | -- | -- |
| Meristemomyces frigidus | ASV_8717 | -- | 2.1 | -- | -- |
| Mycoleptodiscus spp. | ASV_836 | -- | 2.6 | -- | -- |
| Myriangiales NA | ASV_2776 | -- | 2.4 | -- | -- |
| Naganishia diffluens | ASV_4013 | -- | 2.2 | -- | -- |
| Neodevriesiaceae spp. | ASV_2444 | -- | 2.4 | -- | -- |
| Parengyodontium album | ASV_1229 | -- | 2.1 | -- | -- |
| Penicillium spp. | ASV_3151 | -- | 3.1 | -- | -- |
| Peniophorella praetermissa | ASV_3789 | -- | 3.0 | -- | -- |
| Periconia spp. | ASV_3201 | -- | 2.3 | -- | -- |
| Pezizales spp. | ASV_5310 | -- | 2.1 | -- | -- |
| Phaeosphaeriaceae spp. | ASV_3644 | -- | 2.0 | -- | -- |
| Phoma omnivirens | ASV_3406 | -- | 2.2 | -- | -- |
| Pleosporales spp. | ASV_3598 | -- | 2.4 | -- | -- |
| Polyporales spp. | ASV_507 | -- | 2.4 | -- | -- |
| Polyporales spp. | ASV_5005 | -- | 2.3 | -- | -- |
| Ramularia spp. | ASV_7271 | -- | 2.2 | -- | -- |
| Rhodosporidiobolus spp. | ASV_974 | -- | 2.2 | -- | -- |
| Rhodotorula mucilaginosa | ASV_5122 | -- | 2.0 | -- | -- |
| Rutstroemiaceae spp. | ASV_3820 | -- | 2.5 | -- | -- |
| Septoria spp. | ASV_3969 | -- | 2.6 | -- | -- |
| Sistotrema sernanderi | ASV_3730 | -- | 2.1 | -- | -- |
| Sistotremastrum guttuliferum | ASV_1439 | -- | 2.3 | -- | -- |
| Stachybotryaceae spp. | ASV_294 | -- | 2.2 | -- | -- |
| Stereum complicatum | ASV_1394 | -- | 2.2 | -- | -- |
| Stereum complicatum | ASV_4564 | -- | 2.8 | -- | -- |
| Symmetrospora gracilis | ASV_2921 | -- | 3.2 | -- | -- |
| Torula spp. | ASV_4141 | -- | 2.3 | -- | -- |
| Trametes hirsuta | ASV_305 | -- | 3.1 | -- | -- |
| Trechispora stellulata | ASV_4879 | -- | 2.2 | -- | -- |
| Tremellales spp. | ASV_1479 | -- | 2.4 | -- | -- |
| Tyromyces galactinus | ASV_412 | -- | 2.3 | -- | -- |
| Unidentified fungus | ASV_2891 | -- | 2.2 | -- | -- |
| Wallemia spp. | ASV_1270 | -- | 3.3 | -- | -- |

# Text S1 Supplementary Text

## Comparison of UNITE global singletons versus UNITE reference singletons

In approach 1, we observed 57 species with the UNITE reference singletons fungal database. Counterintuitively, the proportion of sequences representing taxa with known $a_w$ requirements for growth was greater when taxonomy was assigned with reference singletons (35%) than when assigned with global singletons (28%) even though the UNITE database with reference singletons contains fewer sequences (~18K taxa) than the database with global singletons (~35K taxa). We attribute this to the fact that there were more taxa unresolved at the species level (i.e. “NA”) when taxonomy was assigned with UNITE global singletons (n=96 taxa) than when assigned with UNITE reference singletons (n=26 taxa).

The taxonomic assignment of the sequences could differ between UNITE databases: 52 species were identified in both UNITE databases, while an additional eight species were found only when taxonomy was assigned with global singletons, and an additional five species only when taxonomy was assigned with reference singletons. Nevertheless, over 65% of the 627 ASVs were identified to the same species between the two databases and another 14% of these 627 ASVs were identified to the same genus but differed at the species-level identification between the two databases. Only 5 taxa (<1% of the ASVs) were identified to different genera by the two fungal databases. The most common example of different species assignments was that many ASVs were identified *as Aspergillus versicolor* using the UNITE fungal database with global singletons but as *A. sydowii* in the database with reference singletons. However, both of these species are considered xerophilic fungi and thus would be included in the targeted xerophilic group. Finally, 20% of the taxa were identified to a species of interest with one database but were unresolved (i.e. “NA”) at the genus or species level with the other database.

In approach 2, 28 were identified in the mycobiome data with reference singletons, representing over 10% of the sequences. As with Approach 1, assigning taxonomy with reference singletons captured slightly more named species in the current dataset than assigning taxonomy with global singletons. Most Group 1 fungi were found in both fungal databases, although *Aspergillus fischeri* and *A. foetidus* were identified only in the database with global singletons, and *A. sclerotiorum*, *Chaetomium globosum*, *Penicillium spinulosum*, and *Trichoderma koningii* were identified only in the database with reference singletons.

| Representation in our dataset of fungi either with known moisture requirements for growth or included in Group 1 ERMI fungi | | | | |
|---|---|---|---|---|
| | Moisture requirements for growth | Number of taxa identified in the literature | Number of those taxa identified in this dataset | Percentage of total sequences in this dataset |
| Fungal database with reference singletons | | | | |
| Hydrophilic | $\geq 0.90\ a_w$ | 18 | 6 | 3.2 |
| Mesophilic | $0.80 \leq a_w < 0.90$ | 61 | 32 | 26.2 |
| Xerophilic | $< 0.80\ a_w$ | 29 | 19 | 5.7 |
| Group 1 | -- | 43 | 28 | 10.4 |

## Bioinformatic scripts in R: Sequence processing of amplicon data

```
### load necessary packages
library("dada2")
library(ShortRead)
library(Biostrings)
library(phyloseq)
library(seqRFLP)
library(ggplot2)
library(decontam); packageVersion("decontam")

### set path
wpath_in <- ".../itsxpress/Winter_itsxpress"
spath_in <- ".../itsxpress/Summer_itsxpress"
#list.files(wpath)
#list.files(spath)

### list input files, with full path names
wpathfilesfull <- sort(list.files(wpath_in , pattern = ".fastq.gz", full.names = TRUE))
spathfilesfull <- sort(list.files(spath_in , pattern = ".fastq.gz", full.names = TRUE))

### get sample names
wpathfiles <- sort(list.files(wpath_in , pattern = ".fastq.gz"))
spathfiles <- sort(list.files(spath_in , pattern = ".fastq.gz"))
w.sample.names <- sapply(strsplit(wpathfiles, "_"), `[`, 1)
s.sample.names <- sapply(strsplit(spathfiles, "_"), `[`, 1)

#optional - check out quality scores
#plotQualityProfile(wpathfilesfull[1:2])

### Filter and trim
wpath_filt <- ".../dada2/winter_filt"
spath_filt <- ".../dada2/summer_filt"
filtWs <- file.path(wpath_filt, basename(wpathfilesfull))
filtSs <- file.path(spath_filt, basename(spathfilesfull))

w.out <- filterAndTrim(wpathfilesfull, filtWs, maxN = 0, maxEE = 2,
   truncQ = 2, minLen = 50, rm.phix = TRUE, compress = TRUE, multithread =
TRUE,verbose=TRUE)
write.table(w.out,".../dada2/w.out.filt.txt",sep="\t",col.names=NA,row.names=TRUE)

s.out <- filterAndTrim(spathfilesfull, filtSs, maxN = 0, maxEE = 2,
   truncQ = 2, minLen = 50, rm.phix = TRUE, compress = TRUE, multithread =
TRUE,verbose=TRUE)
write.table(s.out,".../dada2/s.out.filt.txt",sep="\t",col.names=NA,row.names=TRUE)
```

```
### Learn and view the Error rate
errW <- learnErrors(filtWs, multithread = TRUE)
errS <- learnErrors(filtSs, multithread = TRUE)
#saveRDS(errW,".../dada2/RDSfiles/errW.rds")
#saveRDS(errS,".../dada2/RDSfiles/errS.rds")
#plotErrors(errW, nominalQ=TRUE)
#plotErrors(errS, nominalQ=TRUE)

### Dereplicate
derepWs <- derepFastq(filtWs, verbose = FALSE)
derepSs <- derepFastq(filtSs, verbose = FALSE)

identical(length(derepWs),length(w.sample.names))
identical(length(derepSs),length(s.sample.names))

### Name the derep-class objects by the sample names
names(derepWs) <- w.sample.names
names(derepSs) <- s.sample.names

### Sample Inference
dadaWs <- dada(derepWs, err = errW, multithread = TRUE)
#saveRDS(dadaWs,".../dada2/RDSfiles/dadaWs.rds")
#dadaWs=readRDS(".../dada2/RDSfiles/dadaWs.rds")
dadaSs <- dada(derepSs, err = errS, multithread = TRUE)
#saveRDS(dadaSs,".../dada2/RDSfiles/dadaSs.rds")
#dadaSs=readRDS(".../dada2/RDSfiles/dadaSs.rds")

### Sequence tables and merge
w.seqtab <- makeSequenceTable(dadaWs)
dim(w.seqtab)
s.seqtab <- makeSequenceTable(dadaSs)
dim(s.seqtab)

mergedtab <- mergeSequenceTables(w.seqtab,s.seqtab)

mergedtab.nochim <- removeBimeraDenovo(mergedtab, method="consensus",
multithread=TRUE, verbose=TRUE)
#Identified 2970 bimeras out of 28600 input sequences.
#saveRDS(mergedtab.nochim,".../dada2/RDSfiles/mergedtab.nochim.rds")
#mergedtab.nochim = readRDS(".../dada2/RDSfiles/mergedtab.nochim.rds")
#dim(mergedtab.nochim) [1]   596 25630
#table(nchar(getSequences(mergedtab.nochim)))

### Rename taxonomic id's with generic "ASV_#"
rep_seq=cbind.data.frame(seq=colnames(mergedtab.nochim))
rownames(rep_seq) <- paste("ASV_", 1:length(colnames(mergedtab.nochim)), sep="")
```

```
write.table(rep_seq,".../dada2/RDSfiles/ASVseq.txt",sep="\t",col.names=NA,row.names=TRUE)
rep_seq=cbind.data.frame(seq=colnames(mergedtab.nochim))
names = rownames(rep_seq)
sequences = rep_seq[,1]
df <- data.frame(names,sequences)
df.fasta = dataframe2fas(df, file=".../dada2/RDSfiles/ASVseq.fasta")

mergedtab.nochim.newnames = mergedtab.nochim
colnames(mergedtab.nochim.newnames) <- paste("ASV_",
1:length(colnames(mergedtab.nochim)), sep="")


############### Assign taxonomy - with global singletons *see below for taxonomy with
reference singletons
unite.ref <- ".../UNITE/sh_general_release_dynamic_s_02.02.2019.fasta"  #redid when new
UNITE database came out
#taxa <- assignTaxonomy(mergedtab.nochim, unite.ref, multithread = TRUE, tryRC = TRUE)
###this craashes R, so split then recombine

tm=as.data.frame(t(mergedtab.nochim)) #save as data frame

mergedtab.nochim.split=split(tm,rep(1:ceiling(nrow(tm)/1000),each=1000,length.out=nrow(tm))
) #split into small data frames

## script for writing assignTaxonomy command
# for (i in 1:length(mergedtab.nochim.split)){
	# print(paste0("mergedtab.nochim.split",i,".taxa <-
assignTaxonomy(t(mergedtab.nochim.split$'",i,"'), unite.ref, multithread = TRUE, tryRC =
TRUE)"))
	# }

##run overnight
mergedtab.nochim.split1.taxa <- assignTaxonomy(t(mergedtab.nochim.split$'1'), unite.ref,
multithread = TRUE, tryRC = TRUE)
mergedtab.nochim.split2.taxa <- assignTaxonomy(t(mergedtab.nochim.split$'2'), unite.ref,
multithread = TRUE, tryRC = TRUE)
mergedtab.nochim.split3.taxa <- assignTaxonomy(t(mergedtab.nochim.split$'3'), unite.ref,
multithread = TRUE, tryRC = TRUE)
mergedtab.nochim.split4.taxa <- assignTaxonomy(t(mergedtab.nochim.split$'4'), unite.ref,
multithread = TRUE, tryRC = TRUE)
mergedtab.nochim.split5.taxa <- assignTaxonomy(t(mergedtab.nochim.split$'5'), unite.ref,
multithread = TRUE, tryRC = TRUE)
mergedtab.nochim.split6.taxa <- assignTaxonomy(t(mergedtab.nochim.split$'6'), unite.ref,
multithread = TRUE, tryRC = TRUE)
mergedtab.nochim.split7.taxa <- assignTaxonomy(t(mergedtab.nochim.split$'7'), unite.ref,
multithread = TRUE, tryRC = TRUE)
```

```
mergedtab.nochim.split8.taxa <- assignTaxonomy(t(mergedtab.nochim.split$'8'), unite.ref,
multithread = TRUE, tryRC = TRUE)
mergedtab.nochim.split9.taxa <- assignTaxonomy(t(mergedtab.nochim.split$'9'), unite.ref,
multithread = TRUE, tryRC = TRUE)
mergedtab.nochim.split10.taxa <- assignTaxonomy(t(mergedtab.nochim.split$'10'), unite.ref,
multithread = TRUE, tryRC = TRUE)
mergedtab.nochim.split11.taxa <- assignTaxonomy(t(mergedtab.nochim.split$'11'), unite.ref,
multithread = TRUE, tryRC = TRUE)
mergedtab.nochim.split12.taxa <- assignTaxonomy(t(mergedtab.nochim.split$'12'), unite.ref,
multithread = TRUE, tryRC = TRUE)
mergedtab.nochim.split13.taxa <- assignTaxonomy(t(mergedtab.nochim.split$'13'), unite.ref,
multithread = TRUE, tryRC = TRUE)
mergedtab.nochim.split14.taxa <- assignTaxonomy(t(mergedtab.nochim.split$'14'), unite.ref,
multithread = TRUE, tryRC = TRUE)
mergedtab.nochim.split15.taxa <- assignTaxonomy(t(mergedtab.nochim.split$'15'), unite.ref,
multithread = TRUE, tryRC = TRUE)
mergedtab.nochim.split16.taxa <- assignTaxonomy(t(mergedtab.nochim.split$'16'), unite.ref,
multithread = TRUE, tryRC = TRUE)
mergedtab.nochim.split17.taxa <- assignTaxonomy(t(mergedtab.nochim.split$'17'), unite.ref,
multithread = TRUE, tryRC = TRUE)
mergedtab.nochim.split18.taxa <- assignTaxonomy(t(mergedtab.nochim.split$'18'), unite.ref,
multithread = TRUE, tryRC = TRUE)
mergedtab.nochim.split19.taxa <- assignTaxonomy(t(mergedtab.nochim.split$'19'), unite.ref,
multithread = TRUE, tryRC = TRUE)
mergedtab.nochim.split20.taxa <- assignTaxonomy(t(mergedtab.nochim.split$'20'), unite.ref,
multithread = TRUE, tryRC = TRUE)
mergedtab.nochim.split21.taxa <- assignTaxonomy(t(mergedtab.nochim.split$'21'), unite.ref,
multithread = TRUE, tryRC = TRUE)
mergedtab.nochim.split22.taxa <- assignTaxonomy(t(mergedtab.nochim.split$'22'), unite.ref,
multithread = TRUE, tryRC = TRUE)
mergedtab.nochim.split23.taxa <- assignTaxonomy(t(mergedtab.nochim.split$'23'), unite.ref,
multithread = TRUE, tryRC = TRUE)
mergedtab.nochim.split24.taxa <- assignTaxonomy(t(mergedtab.nochim.split$'24'), unite.ref,
multithread = TRUE, tryRC = TRUE)
mergedtab.nochim.split25.taxa <- assignTaxonomy(t(mergedtab.nochim.split$'25'), unite.ref,
multithread = TRUE, tryRC = TRUE)
mergedtab.nochim.split26.taxa <- assignTaxonomy(t(mergedtab.nochim.split$'26'), unite.ref,
multithread = TRUE, tryRC = TRUE)

save.image(".../dada2/RDSfiles/dada-fungi.RData")
#readRDS(".../dada2/RDSfiles/dada-fungi.RData")

# for (i in 1:length(mergedtab.nochim.split)){
	# print(paste0("mergedtab.nochim.split",i,".taxa"))
	# }
```

```
### merge the separate taxonomy files
tax.comb=rbind(mergedtab.nochim.split1.taxa,
            mergedtab.nochim.split2.taxa,
            mergedtab.nochim.split3.taxa,
            mergedtab.nochim.split4.taxa,
            mergedtab.nochim.split5.taxa,
            mergedtab.nochim.split6.taxa,
            mergedtab.nochim.split7.taxa,
            mergedtab.nochim.split8.taxa,
            mergedtab.nochim.split9.taxa,
            mergedtab.nochim.split10.taxa,
            mergedtab.nochim.split11.taxa,
            mergedtab.nochim.split12.taxa,
            mergedtab.nochim.split13.taxa,
            mergedtab.nochim.split14.taxa,
            mergedtab.nochim.split15.taxa,
            mergedtab.nochim.split16.taxa,
            mergedtab.nochim.split17.taxa,
            mergedtab.nochim.split18.taxa,
            mergedtab.nochim.split19.taxa,
            mergedtab.nochim.split20.taxa,
            mergedtab.nochim.split21.taxa,
            mergedtab.nochim.split22.taxa,
            mergedtab.nochim.split23.taxa,
            mergedtab.nochim.split24.taxa,
            mergedtab.nochim.split25.taxa,
            mergedtab.nochim.split26.taxa)

identical(rownames(tax.comb),colnames(mergedtab.nochim))

saveRDS(tax.comb,".../dada2/RDSfiles/taxa.rds")
#tax.comb = readRDS(".../dada2/RDSfiles/taxa.rds")

## rename taxa names with generic ASV_# names
tax.comb.temp=tax.comb
seq <- rownames(tax.comb.temp)
rownames(tax.comb.temp) <- paste("ASV_", 1:length(rownames(tax.comb.temp)), sep="")
tax.comb.full=cbind(seq,tax.comb.temp)
#write.table(tax.comb.full,".../dada2/RDSfiles/tax.comb.full.txt",sep="\t",col.names=NA,row.na
mes=TRUE)

### Create phyloseq object
ps.pre1 = phyloseq(otu_table(mergedtab.nochim.newnames,taxa_are_rows=FALSE),
tax_table(tax.comb.temp))
#saveRDS(ps.pre1,".../dada2/RDSfiles/mergedtab.nochim+taxa.rds")
#ps.pre1 = readRDS(".../dada2/RDSfiles/mergedtab.nochim+taxa.rds")
```

```
### Add metadata
ps.pre1 = readRDS(".../dada2/RDSfiles/mergedtab.nochim+taxa.rds")
meta.in=read.table(".../Metadata/samples_in_final_table_withmeta.txt",header=TRUE)
ps.pre2=merge_phyloseq(ps.pre1,sample_data(meta.in))
saveRDS(ps.pre2,".../dada2/RDSfiles/mergedtab.nochim+taxa+meta.rds")
#ps.pre2 = readRDS(".../dada2/RDSfiles/mergedtab.nochim+taxa+meta.rds")


############### Assign taxonomy with global singletons
unite.ref <- "~/Documents/Software/UNITE/sh_general_release_dynamic_02.02.2019.fasta"
#redid when new UNITE database came out
#taxa <- assignTaxonomy(mergedtab.nochim, unite.ref, multithread = TRUE, tryRC = TRUE)
#this craashes R

tm=as.data.frame(t(mergedtab.nochim))

mergedtab.nochim.split=split(tm,rep(1:ceiling(nrow(tm)/1000),each=1000,length.out=nrow(tm))
)

# for (i in 1:length(mergedtab.nochim.split)){
	# print(paste0("mergedtab.nochim.split",i,".taxa <-
assignTaxonomy(t(mergedtab.nochim.split$'",i,"'), unite.ref, multithread = TRUE, tryRC =
TRUE)"))
	# }

##run overnight
mergedtab.nochim.split1.taxa <- assignTaxonomy(t(mergedtab.nochim.split$'1'), unite.ref,
multithread = TRUE, tryRC = TRUE)
mergedtab.nochim.split2.taxa <- assignTaxonomy(t(mergedtab.nochim.split$'2'), unite.ref,
multithread = TRUE, tryRC = TRUE)
mergedtab.nochim.split3.taxa <- assignTaxonomy(t(mergedtab.nochim.split$'3'), unite.ref,
multithread = TRUE, tryRC = TRUE)
mergedtab.nochim.split4.taxa <- assignTaxonomy(t(mergedtab.nochim.split$'4'), unite.ref,
multithread = TRUE, tryRC = TRUE)
mergedtab.nochim.split5.taxa <- assignTaxonomy(t(mergedtab.nochim.split$'5'), unite.ref,
multithread = TRUE, tryRC = TRUE)
mergedtab.nochim.split6.taxa <- assignTaxonomy(t(mergedtab.nochim.split$'6'), unite.ref,
multithread = TRUE, tryRC = TRUE)
mergedtab.nochim.split7.taxa <- assignTaxonomy(t(mergedtab.nochim.split$'7'), unite.ref,
multithread = TRUE, tryRC = TRUE)
mergedtab.nochim.split8.taxa <- assignTaxonomy(t(mergedtab.nochim.split$'8'), unite.ref,
multithread = TRUE, tryRC = TRUE)
mergedtab.nochim.split9.taxa <- assignTaxonomy(t(mergedtab.nochim.split$'9'), unite.ref,
multithread = TRUE, tryRC = TRUE)
```

```
mergedtab.nochim.split10.taxa <- assignTaxonomy(t(mergedtab.nochim.split$'10'), unite.ref,
multithread = TRUE, tryRC = TRUE)
mergedtab.nochim.split11.taxa <- assignTaxonomy(t(mergedtab.nochim.split$'11'), unite.ref,
multithread = TRUE, tryRC = TRUE)
mergedtab.nochim.split12.taxa <- assignTaxonomy(t(mergedtab.nochim.split$'12'), unite.ref,
multithread = TRUE, tryRC = TRUE)
mergedtab.nochim.split13.taxa <- assignTaxonomy(t(mergedtab.nochim.split$'13'), unite.ref,
multithread = TRUE, tryRC = TRUE)
mergedtab.nochim.split14.taxa <- assignTaxonomy(t(mergedtab.nochim.split$'14'), unite.ref,
multithread = TRUE, tryRC = TRUE)
mergedtab.nochim.split15.taxa <- assignTaxonomy(t(mergedtab.nochim.split$'15'), unite.ref,
multithread = TRUE, tryRC = TRUE)
mergedtab.nochim.split16.taxa <- assignTaxonomy(t(mergedtab.nochim.split$'16'), unite.ref,
multithread = TRUE, tryRC = TRUE)
mergedtab.nochim.split17.taxa <- assignTaxonomy(t(mergedtab.nochim.split$'17'), unite.ref,
multithread = TRUE, tryRC = TRUE)
mergedtab.nochim.split18.taxa <- assignTaxonomy(t(mergedtab.nochim.split$'18'), unite.ref,
multithread = TRUE, tryRC = TRUE)
mergedtab.nochim.split19.taxa <- assignTaxonomy(t(mergedtab.nochim.split$'19'), unite.ref,
multithread = TRUE, tryRC = TRUE)
mergedtab.nochim.split20.taxa <- assignTaxonomy(t(mergedtab.nochim.split$'20'), unite.ref,
multithread = TRUE, tryRC = TRUE)
mergedtab.nochim.split21.taxa <- assignTaxonomy(t(mergedtab.nochim.split$'21'), unite.ref,
multithread = TRUE, tryRC = TRUE)
mergedtab.nochim.split22.taxa <- assignTaxonomy(t(mergedtab.nochim.split$'22'), unite.ref,
multithread = TRUE, tryRC = TRUE)
mergedtab.nochim.split23.taxa <- assignTaxonomy(t(mergedtab.nochim.split$'23'), unite.ref,
multithread = TRUE, tryRC = TRUE)
mergedtab.nochim.split24.taxa <- assignTaxonomy(t(mergedtab.nochim.split$'24'), unite.ref,
multithread = TRUE, tryRC = TRUE)
mergedtab.nochim.split25.taxa <- assignTaxonomy(t(mergedtab.nochim.split$'25'), unite.ref,
multithread = TRUE, tryRC = TRUE)
mergedtab.nochim.split26.taxa <- assignTaxonomy(t(mergedtab.nochim.split$'26'), unite.ref,
multithread = TRUE, tryRC = TRUE)

save.image(".../dada2/RDSfiles/dada-fungi-unite2019-noS.RData")
#readRDS(".../dada2/RDSfiles/dada-fungi-unite2019-noS.RData")

# for (i in 1:length(mergedtab.nochim.split)){
	# print(paste0("mergedtab.nochim.split",i,".taxa"))
	# }

tax.comb=rbind(mergedtab.nochim.split1.taxa,
          mergedtab.nochim.split2.taxa,
          mergedtab.nochim.split3.taxa,
          mergedtab.nochim.split4.taxa,
```

```
         mergedtab.nochim.split5.taxa,
         mergedtab.nochim.split6.taxa,
         mergedtab.nochim.split7.taxa,
         mergedtab.nochim.split8.taxa,
         mergedtab.nochim.split9.taxa,
         mergedtab.nochim.split10.taxa,
         mergedtab.nochim.split11.taxa,
         mergedtab.nochim.split12.taxa,
         mergedtab.nochim.split13.taxa,
         mergedtab.nochim.split14.taxa,
         mergedtab.nochim.split15.taxa,
         mergedtab.nochim.split16.taxa,
         mergedtab.nochim.split17.taxa,
         mergedtab.nochim.split18.taxa,
         mergedtab.nochim.split19.taxa,
         mergedtab.nochim.split20.taxa,
         mergedtab.nochim.split21.taxa,
         mergedtab.nochim.split22.taxa,
         mergedtab.nochim.split23.taxa,
         mergedtab.nochim.split24.taxa,
         mergedtab.nochim.split25.taxa,
         mergedtab.nochim.split26.taxa)

identical(rownames(tax.comb),colnames(mergedtab.nochim))

saveRDS(tax.comb,".../dada2/RDSfiles/taxa-unite2019-noS.rds")
#tax.comb=readRDS(".../dada2/RDSfiles/taxa-unite2019-noS.rds")

tax.comb.temp=tax.comb
seq <- rownames(tax.comb.temp)
rownames(tax.comb.temp) <- paste("ASV_", 1:length(rownames(tax.comb.temp)), sep="")
tax.comb.full=cbind(seq,tax.comb.temp)
write.table(tax.comb.full,".../dada2/RDSfiles/tax.comb.full-
noS.txt",sep="\t",col.names=NA,row.names=TRUE)

### Create phyloseq object
ps = phyloseq(otu_table(mergedtab.nochim.newnames,taxa_are_rows=FALSE),
tax_table(tax.comb.temp))
#saveRDS(ps,".../dada2/RDSfiles/mergedtab.nochim+taxa+unite2019-noS.rds")
#ps_noS=readRDS(".../dada2/RDSfiles/mergedtab.nochim+taxa+unite2019-noS.rds")


## Add metadata
ps_noS=readRDS(".../dada2/RDSfiles/mergedtab.nochim+taxa+unite2019-noS.rds")
meta.in=read.table(".../Metadata/samples_in_final_table_withmeta.txt",header=TRUE)
ps_noS.pre2=merge_phyloseq(ps_noS,sample_data(meta.in))
```

```
saveRDS(ps_noS.pre2,".../dada2/RDSfiles/mergedtab.nochim+taxa+unite2019-noS+meta-
nometa.rds")

################## looking at potential contaminants using the decontam package,
# following: https://benjjneb.github.io/decontam/vignettes/decontam_intro.html

#add new metadata about sample or control
sample_data(ps)$Sample_or_Control <-
ifelse(sample_data(ps)$indoor_outdoor=="blank"|sample_data(ps)$indoor_outdoor=="control","
Control","Sample")

#inspect library sizes
df <- as.data.frame(sample_data(ps)) # Put sample_data into a ggplot-friendly data.frame
df$LibrarySize <- sample_sums(ps)
df <- df[order(df$LibrarySize),]
df$Index <- seq(nrow(df))
ggplot(data=df, aes(x=Index, y=LibrarySize, color=Sample_or_Control)) + geom_point()

##too many missing fungal biomass estimates to use frequency approach, so doing prevalence
approach
sample_data(ps)$is.neg <- sample_data(ps)$Sample_or_Control == "Control"
contamdf.prev <- isContaminant(ps, method="prevalence", neg="is.neg")
table(contamdf.prev$contaminant)
#FALSE  TRUE
#25609    21
head(which(contamdf.prev$contaminant))
#[1]   5  16  51  91  96 125
contamdf.prev05 <- isContaminant(ps, method="prevalence", neg="is.neg", threshold=0.5)
table(contamdf.prev05$contaminant)
#FALSE  TRUE
#25515   115
head(which(contamdf.prev05$contaminant))
#[1]  5 16 23 51 91 96

ps.pa <- transform_sample_counts(ps, function(abund) 1*(abund>0))
ps.pa.neg <- prune_samples(sample_data(ps.pa)$Sample_or_Control == "Control", ps.pa)
ps.pa.pos <- prune_samples(sample_data(ps.pa)$Sample_or_Control == "Sample", ps.pa)
# Make data.frame of prevalence in positive and negative samples
df.pa <- data.frame(pa.pos=taxa_sums(ps.pa.pos), pa.neg=taxa_sums(ps.pa.neg),
                    contaminant=contamdf.prev$contaminant)
ggplot(data=df.pa, aes(x=pa.neg, y=pa.pos, color=contaminant)) + geom_point() +
  xlab("Prevalence (Negative Controls)") + ylab("Prevalence (True Samples)")

df.pa <- data.frame(pa.pos=taxa_sums(ps.pa.pos), pa.neg=taxa_sums(ps.pa.neg),
                    contaminant=contamdf.prev05$contaminant)
ggplot(data=df.pa, aes(x=pa.neg, y=pa.pos, color=contaminant)) + geom_point() +
```

```
  xlab("Prevalence (Negative Controls)") + ylab("Prevalence (True Samples)")

# remove contaminant taxa and control samples from phyloseq object with taxonomy with global singletons
ps.noncontam <- prune_taxa(!contamdf.prev05$contaminant, ps)
ps.noncontam <- subset_samples(ps.noncontam,Sample_or_Control == "Sample") #remove the control samples
ps.noncontam

# remove contaminant taxa and control samples from phyloseq object with taxonomy without reference singletons
ps_noS.noncontam <- prune_taxa(!contamdf.prev05$contaminant, ps_noS)
ps_noS.noncontam <- subset_samples(ps_noS.noncontam,Sample_or_Control == "Sample")
ps_noS.noncontam
```

## Bioinformatic scripts in R: Isolating taxa of interest from a phyloseq object

```
# load required packages without warnings,messages
suppressWarnings(suppressMessages(library(phyloseq)))
suppressWarnings(suppressMessages(library(tidyr)))
suppressWarnings(suppressMessages(library(dplyr)))

# import phyloseq object, called 'ps' for simplicity
ps = readRDS("~/.../phyloseq.rds")

# read in table of fungi with moisture requirements for growth:
# column 1: named "taxa", species name, separated by underscore so there's no space, e.g,
"Sistotrema_brinkmannii"
# column 2: named "mean_min_aw"
# column 3: named "min_aw"; note this column wasn't used in processing
target.aw=read.table("~/.../moist_req_fungi.txt",header=TRUE)

# create fungal groups based on moisture requirements for growth; see how many taxa are in
each group
hydro_level = 0.90
xero_level = 0.80

target.hydro = target.aw[target.aw$mean_min_aw >= hydro_level,]; dim(target.hydro)
target.meso = target.aw[target.aw$mean_min_aw >= xero_level & target.aw$mean_min_aw <
hydro_level,]; dim(target.meso)
target.xero = target.aw[target.aw$mean_min_aw < xero_level,]; dim(target.xero)

# create data frame of taxonomic names in phyloseq to work with, in order to get taxonomic
identity in Genus_species format
gs=as.data.frame(tax_table(ps)[,6:7]) # dataframe of taxa in ps, this step takes a while depending
on the size of the taxonomy table
gs[,"Genus_species"]=paste0(gsub("g__","\\1",gs$Genus),"_",gsub("s__","\\1",gs$Species))
#create new column drawing on the existing columns, in Genus_species format

# get new phyloseq object with just the targeted list of all taxa of known moisture requirements
gs.target.aw=gs[(gs[,"Genus_species"] %in% target.aw$taxa),] #get subsetted data frame of
those ASVs in the targeted list
ps.target.aw=prune_taxa(as.vector(rownames(gs.target.aw)),ps) #pruning the phyloseq object to
the targeted list

# get new phyloseq object with the individual groups of fungi

# hydrophilic fungi
gs.target.hydro=gs[(gs[,"Genus_species"] %in% target.hydro$taxa),] #get subsetted data frame
of those ASVs in the targeted list
```

ps.target.hydro=prune_taxa(as.vector(rownames(gs.target.hydro)),ps) #get new phyloseq table with just the targeted list

# mesophilic fungi
gs.target.meso=gs[(gs[,"Genus_species"] %in% target.meso$taxa),] #get subsetted data frame of those ASVs in the targeted list
ps.target.meso=prune_taxa(as.vector(rownames(gs.target.meso)),ps) #get new phyloseq table with just the targeted list

# xerophilic fungi
gs.target.xero=gs[(gs[,"Genus_species"] %in% target.xero$taxa),] #get subsetted data frame of those ASVs in the targeted list
ps.target.xero=prune_taxa(as.vector(rownames(gs.target.xero)),ps) #get new phyloseq table with just the targeted list
